# Discovery and validation of *ASG1* as a novel determinant of NaCl tolerance in the yeast *Saccharomyces cerevisiae* through iterative crossing

**DOI:** 10.1093/g3journal/jkaf254

**Published:** 2025-10-28

**Authors:** Gašper Žun, Uroš Petrovič

**Affiliations:** Department of Molecular and Biomedical Sciences, Jožef Stefan Institute, Jamova cesta 39, Ljubljana 1000, Slovenia; Biotechnical Faculty, Department of Biology, University of Ljubljana, Večna pot 111, Ljubljana 1000, Slovenia; Department of Molecular and Biomedical Sciences, Jožef Stefan Institute, Jamova cesta 39, Ljubljana 1000, Slovenia; Biotechnical Faculty, Department of Biology, University of Ljubljana, Večna pot 111, Ljubljana 1000, Slovenia

**Keywords:** yeast *Saccharomyces cerevisiae*, salt tolerance, iterative crossing, QTL mapping, allele swapping, *ASG1* gene

## Abstract

To dissect the genetic basis of quantitative traits, generation of numerous haploid segregants with diverse genotypes and phenotypes from heterozygous parental strains is a powerful approach. To identify quantitative trait loci (QTLs) associated with NaCl salt tolerance, we employed an iterative crossing strategy using parental strains with contrasting phenotypes. Whole-genome sequencing of selected individual offspring with the most extreme trait value from each generation as well as of the pools of segregants under extreme salt conditions enabled QTL mapping and identification of candidate causative variants. Their effects on phenotypic variation were quantified through a genome-wide screen of generation-dependent reduction of the causative loci and by allele swapping procedure of the putative quantitative trait genes in isogenic strain backgrounds. A combination of these complementary approaches enabled assessment of the causal loci with the strongest effect. We thus confirmed the causative role of the *ENA* locus, and proposed an additional contribution of the *ASG1* gene in NaCl salt tolerance. Asg1 (Activator of Stress Genes 1) has been proposed as a transcriptional regulator of genes involved in lipid metabolism and various stress responses. Previous large-scale studies have indicated that Asg1 could have a negative effect on NaCl tolerance in *S. cerevisiae*. The results of our study confirm that prediction and further elucidate its previously uncharacterized negative role in NaCl stress adaptation. Our species-wide association analysis supports a universal contribution of *ASG1* gene to NaCl tolerance, which had been masked by the dominant influence of the *ENA* locus in *S. cerevisiae*.

## Graphical Abstract

**Graphical Abstract:**
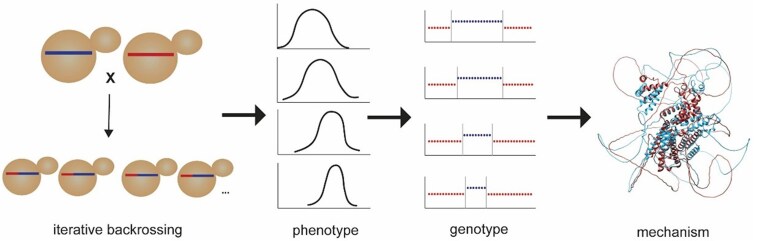

## Introduction

The central problem of genetics remains finding the exact sources of traits' variability by uncovering their genetic architecture (Liti and Louis 2012; Hallin et al. 2016; Bloom et al. 2019), whose complexity can be highly dependent on genetic background (Hou et al. 2016, 2018). Generally, the mode of inheritance divides the traits into monogenic and complex (i.e. polygenic) (Hou et al. 2016) with the former being controlled predominantly by one gene and the latter being partly influenced by additional genetic factors (Hou et al. 2016, 2018). Polygenic trait inheritance is reflected in a unimodal (normal) population distribution due to contribution of multiple (although finite (Jakobson and Jarosz 2019)) number of loci to the genetic value inheritance potential (Hou et al. 2016; Jakobson and Jarosz 2019). To dissect quantitative traits by uncovering causative variants with high confidence, even those with small contribution, yeast *Saccharomyces cerevisiae* serves as a powerful tool as it enables creating a large segregant pool by sporulation of a heterozygous diploid (Ehrenreich et al. 2010; Parts et al. 2011; Cubillos et al. 2013; Wang and Kruglyak 2014; Meijnen et al. 2016; Jakobson and Jarosz 2019). The resulting pool of segregants, genetically diversified by meiotic recombination (Liu et al. 2019) and the following independent chromosome segregation (Mancera et al. 2008; Laureau et al. 2016), thus contains a highly related, yet phenotypically variable set of haploid individuals (Mancera et al. 2008; Laureau et al. 2016). Certainty and resolution of the subsequent quantitative trait loci (QTL) assessment depend on the size and heterogeneity of the analyzed population (Ehrenreich et al. 2010; Meijnen et al. 2016; Pačnik et al. 2021). By increasing the frequency of recombination, i.e. by intercrossing or inbreeding (Liti and Louis 2012; Cubillos et al. 2013; Treusch et al. 2015), the size of the causative loci is narrowed (Taşan et al. 2015; She and Jarosz 2018; Jakobson et al. 2019; Jakobson and Jarosz 2019). However, proposed causative variants are rarely validated through biologically relevant assays and are typically supported only by QTL probability estimates (She and Jarosz 2018; Sirr et al. 2018; Pačnik et al. 2021).

QTL mapping (Ehrenreich et al. 2010), conceptually related to genome-wide association studies (GWAS), is used to identify genomic regions associated with trait variation. It relies on progeny derived from controlled, defined crosses that generate recombination and enable the mapping of causal loci. One possible approach considers a trait such as tolerance against an applied selective pressure where genotype characteristics of the tolerant subpopulation are compared to the entire progeny (Ehrenreich et al. 2010; Parts et al. 2011; Cubillos et al. 2013; Wang and Kruglyak 2014; Meijnen et al. 2016; Pačnik et al. 2021). For instance, yeast *S. cerevisiae* generally does not tolerate NaCl as well as some other yeast species (Gunde-Cimerman et al. 2000; Pontes et al. 2019). The presence of NaCl triggers two distinctive responses: osmotic stress and Na^+^ toxicity (Tamás and Hohmann 2007). The major pathway in *S. cerevisiae* to adapt to osmotic stress is regulated by the high-osmolarity glycerol (HOG) pathway, which is a mitogen-activated protein kinase pathway (Van Wuytswinkel et al. 2000; Hohmann 2002; Tamás and Hohmann 2007; Treusch et al. 2015). Sensors on the plasma membrane recognize hyperosmotic conditions, which in response cause phosphorylation of the Hog1 kinase (Hohmann 2002). Activated Hog1 translocates into the nucleus where it primarily acts as a transcription factor to activate osmoprotective glycerol synthesis (Van Wuytswinkel et al. 2000; Hohmann 2002). Activated Hog1 additionally accelerates transcription of other response genes, for instance Exitus NAtru (*ENA*) cluster (Tamás and Hohmann 2007; Warringer et al. 2011; Treusch et al. 2015) which is transcribed after inactivation of its CREBP repressor (Hohmann 2002). These genes encode pumps that facilitate efflux of Na^+^ and Li^+^ ions to establish salt tolerance (Tamás and Hohmann 2007; Warringer et al. 2011; Fay 2013; Treusch et al. 2015; Hou et al. 2018). The overall response through HOG pathway is not dependent on Na^+^ toxicity, but rather on reduced water activity, as similar effects have been observed for K^+^ and sorbitol (Van Wuytswinkel et al. 2000). Elevated Na^+^ specifically triggers calcineurin-signalling pathway whereby Na^+^ causes intracellular release of Ca^2+^ that in turn activates calcineurin phosphatase. The phosphatase causes activation of transcriptional factors that induce transcription of P-type ATPase pumps, which regulate homeostasis of small cations (Hohmann 2002).

In *S. cerevisiae*, the level of NaCl salt tolerance is largely determined by a single major *ENA* locus, to such an extent that in some genetic backgrounds it has even been categorized as a monogenic trait (Anderson et al. 2010; Hou et al. 2016, 2018; Sirr et al. 2018). We hypothesized that in other genetic backgrounds its polygenic nature (Hou et al. 2016) is caused by an involvement of multiple factors either directly in the pathways for Na^+^ rescue or indirectly through general or osmotic stress response (Posas et al. 2000; Rep et al. 2000; Hohmann 2002; Hirasawa et al. 2006) and through energy metabolism (Posas et al. 2000; Rep et al. 2000; Hirasawa et al. 2006). For these reasons, and given the generally low salt tolerance of *S. cerevisiae*, we aimed to identify additional transgressive variants associated with NaCl salt tolerance using QTL mapping approach in the first step, and then to discover and evaluate the contribution of novel quantitative trait genes (QTGs). We monitored the trait distribution through several generations of iterative backcrossing of the directed evolution to provide insight into the causative variants' contribution to trait variation. Finally, variants of potential QTGs within respective QTLs with the strongest impact were assessed within otherwise isogenic strain backgrounds to provide a quantitative evaluation.

## Methods

### Strains and media

The source *S. cerevisiae* strain used in crossing and sporulation experiments of the synthetic genetic array analysis was Y7092 (*MATα can1Δ::STE2pr-Sp_his5 lyp1Δ ura3Δ0 leu2Δ0 his3Δ1 met15Δ0*) (Tong and Boone 2006). It is a derivative of S288C reference *S. cerevisiae* strain. In later steps of the experiment, a simpler version of the same genetic reference background was introduced, namely BY4742 (*MATα leu2Δ0 lys2Δ0 ura3Δ0*) (Goffeau et al. 1996; Brachmann et al. 1998) and denoted throughout this study as BY. The other parental *S. cerevisiae* strain used in crossing procedure was prototrophic CEN.PK113-7D (*MATa MAL2-8c SUC2*) (Daran-Lapujade et al. 2003; Nijkamp et al. 2012; Salazar et al. 2017) which is denoted throughout this study as CEN.PK. First, we prepared the homozygous diploid form of the source CEN.PK113-7D strain that was later sporulated to generate haploids of both mating types. Second, both haploid forms of CEN.PK (*MATa*, *MATα*) and BY4742 were subjected to *HIS3* gene deletion (Supplementary Table 1 in Supplementary File S1) to make them suitable for the crossing and sporulation procedure.

Marker-generated deletions (Janke et al. 2004; Komor et al. 2017) of selected genes and loci were assisted by KanMX resistance gene incorporation. The cassette was amplified by oligonucleotides (Macrogen, South Korea) that possessed overhangs equal to sequences of at least 45 bp up- and downstream of the targeted gene by Q5 Hot Start High-Fidelity DNA polymerase (#M0493L, New England Biolabs, USA). Transformation of this linear donor dsDNA led to target gene deletion with KanMX cassette incorporation as the homology-like sequences that overlapped with the target allowed for recombination by homology-directed repair (HDR). Transformants were selected on YPD G-418 medium and successful deletions were PCR-verified.

Marker-free deletions or allele swaps within beforehand marker-generated deletions were facilitated by CRISPR-Cas9-generated DSB within the target region (Supplementary Table 2 and Supplementary Table 3 in Supplementary File S1). The insertion constructs were PCR-amplified and homology-like sequences that overlapped with the target locus were attached by primers overhangs. Co-transformation of Cas9, gRNA and DNA donor template facilitated insertion within the target locus by HDR.

YPD medium was composed of yeast extract (#1702, Conda, Spain), peptone (#82303, Fluka, Germany), and dextrose. Pre-sporulation medium was composed of yeast extract, peptone and KOAc. Sporulation medium was composed of yeast extract, KOAc, glucose and defined amino acids composition as indicated in Supplementary Table 4 in Supplementary File S1 (Pačnik et al. 2021). Spore-selection medium was composed of YNB medium without amino acids (yeast nitrogen base; #CYN0405, Formedium, England), glucose and defined amino acids composition as indicated in Supplementary Table 5 in Supplementary File S1 (Tong and Boone 2006). Antibiotic nourseothricin (clonNat; #AB-102L, Jena Bioscience, Germany) was added to the final concentration of 100 mg/l, antibiotic G-418 disulfate (#G4185, Formedium, England) to 500 mg/l, and antibiotic hygromycin B (Hyg; #HYG5000, Formedium, England) to 200 mg/ml. Counter-selection markers L-canavanine (#C9758, Sigma Aldrich, Germany) and S-(2-Aminoethyl)-L-cysteine (thialysine; #A2636, Sigma Aldrich, Germany) were added to the spore selection medium both to 60 mg/l.

### Plasmid construction

gRNA expression plasmid p426 (#43803, Addgene) was adapted from DiCarlo *et al.* (DiCarlo et al. 2013). For its general application, the auxotrophic complementation marker was replaced by a dominant selection marker KanMX (Pačnik et al. 2021) or HygMX (Supplementary Table 6 in Supplementary File S1). The designed single crRNA target sequence to be expressed under the SNR52 promoter in tandem with tracrRNA was incorporated by the Gibson assembly (#E2611L, New England Biolabs, USA). In this case, the backbone of the initial plasmid was PCR-amplified with two pairs of overlapping oligonucleotides, where one pair had 20 nt overhangs encoding the crRNA sequence (Žun et al. 2022). The target sequences for the gRNAs were selected using the Benchling tool (Hsu et al. 2014; Doench et al. 2016) according to their specificity (off-target score (Hsu et al. 2014), 100%) and activity (on-target score (Doench et al. 2016), >60).

Plasmids (Supplementary Table 7 in Supplementary File S1) were amplified by bacteria *Escherichia coli* DH5α strain, for which transformation by a standard heat-shock protocol was used (Froger and Hall 2007).

### Yeast transformation

CRISPR-Cas9 endonuclease experiment was performed similarly as described by DiCarlo *et al.* (DiCarlo et al. 2013): SpCas9 endonuclease was firstly expressed from a low-copy CEN6/ARS4 p414 plasmid (#43802, Addgene) with clonNat selection under a strong constitutive TEF1 promoter. gRNA was expressed under the SNR52 promoter from sequentially transformed high-copy 2μ gRNA expression plasmid p426 with G-418 or hygromycin selection. Yeast transformation was performed according to the standard chemical protocol with lithium acetate-ssDNA-PEG as described in Gietz *et al.* (Gietz and Schiestl 2007 ): cells from an overnight culture were grown for four generation times (OD_600_ 0.05 to 0.5) after the dilution into fresh liquid medium. Cells from 10 ml of such freshly grown culture were harvested and prepared chemically competent by washing with lithium acetate. Heat shock at 42 °C for 30 min was performed after the incubation at 30 °C for 1 h, followed by final incubation at 30 °C for 2 h. In the first step, *S. cerevisiae* strains were transformed with the Cas9 expression plasmid and selected on solid agar YPD clonNat plates. In the second step, the selected transformants were co-transformed with 200 ng of the gRNA expression plasmid and 5 μg of linear donor. Selection of two-plasmid transformants was achieved on solid agar YPD clonNat G-418 or YPD clonNat Hyg plates after 3 d of incubation at 30 °C.

### Yeast crossing and sporulation

Haploid *S. cerevisiae* strains with opposite mating types were crossed on a solid YPD agar plate. After 1 d of growth, cells from the center of the cross were resuspended in the YPD liquid medium, diluted, and spread on a new YPD agar plate. Diploid cells in a colony were confirmed by mating type PCR (Supplementary Table 1 in Supplementary File S1) after 2 d of growth.

To sporulate a heterozygous diploid *S. cerevisiae* strain, first, a colony was grown overnight in YPD liquid medium in a test tube with vigorous shaking. After washing the harvested culture with water, the cells were resuspended in a pre-sporulation liquid medium, with KOAc as a source of carbon and peptone as a source of nitrogen. They were grown in a test tube for 3 d at 30 °C with vigorous shaking. After washing the harvested culture from a pre-sporulation medium with water, the cells were resuspended in a sporulation medium, with KOAc and a trace of glucose as a source of carbon and amino acids selection as a source of nitrogen (see the “Strains and media” section). The diploid cells were left to sporulate for 7 d at 20 °C at 60 rpm in a shaking flask. The mixture of sporulated and unsporulated cells was harvested and treated by lyticase (#L2524, Sigma Aldrich) to degrade cell walls. The released segregants were finally selected either on solid agar plates or in liquid medium lacking histidine, arginine and lysine, but with their toxic analogs canavanine and thialysine after two days of growth (see the “Strains and media” section). The composition allowed to select haploids of only one mating type to prevent repeated diploid creation.

### Phenotyping

Individual segregants were transferred into a 384-well plate. The array was transferred onto rectangular transparent plates (#242811, Nunc OmniTray, Thermo Fisher, USA) with solid YPD medium. The transfer of the matrix array from a solid medium onto test plates was performed by Cartesian manipulator (custom-made by Adept Plus, Slovenia) with floating pins (#AFIX384FP3 and #FP3N, V&P Scientific, USA). End-point segregant phenotyping was performed once per day after the inoculation by transmissive image acquisition by Epson Perfection V700 Photo Scanner. End-point colony sizes were extracted by HT Colony Grid Analyzer from the acquired images (Collins et al. 2006) and statistically evaluated (Mattiazzi et al. 2010) by normalization within and between test plates, as follows:


$$\mathrm{relative}\;\mathrm{NaCl}\;\mathrm{tolerance}=\;\frac{\left(\frac{\mathrm{tes}t_{\mathrm{NaCl}}}{\bar{\mathrm{referenc}e_{\mathrm{NaCl}}}}\right)}{\left(\frac{\mathrm{tes}t_{\mathrm{YPD}}}{\bar{\mathrm{referenc}e_{\mathrm{YPD}}}}\right)}$$

Test colony sizes within control YPD plates and testing NaCl plates were first normalized to the growth of the reference BY strain. Subsequent between plates normalization was performed by comparison of the growth in testing NaCl conditions relative to the reference conditions without NaCl. The resulting relative NaCl tolerance was equal to 1 if the testing strain was equally tolerant as the reference strain, whereas lower or higher values corresponded to lower or higher tolerances as the reference strain, respectively. Final distributions of NaCl salt tolerance were plotted in R, where relative NaCl tolerances were distributed into classes of size 0.05 with the reference BY strain corresponding to the 20th class. The shape (i.e. modality) of the empirical frequency distributions was characterized by fitting two-component Gaussian mixture model (GMM) densities using the expectation-maximization algorithm in R. Model comparison, as a measure of goodness of fit, was performed using the Bayesian information criterion (BIC), which penalizes model complexity. A fitted GMM was considered strongly supported only if the BIC difference between competing models exceeded 100 units, ensuring robustness against noise and preventing the identification of minor multimodality. In each generation of crossing, the segregant with the most extreme trait value was selected at higher NaCl concentrations (i.e. ≥1.50 M), while also exhibiting a stable phenotype at other high salt concentrations and ranking among the most salt-tolerant strains.

We acquired quantitative growth parameters in high throughput manner by implementation of Pyphe soft- and hardware (Kamrad et al. 2020), a Python tool for microbial phenotyping. *S. cerevisiae* source wild-type strains, their deletion and swap isogenic alternatives were replicated onto solid YPD agar rectangular transparent plates supplemented with NaCl as described above. Their growth was monitored by continuous image acquisition every 10 mins by Epson Perfection V700 Photo Scanner in the controlled environment of the incubator at 30 °C, coordinated by Pyphe scan-timecourse tool. Growth values, i.e. colony sizes, were extracted from a series of images by the Pyphe quantify tool. By fitting a growth curve to the time series data all the quantitative growth parameters were estimated by the Pyphe growthcurves tool. The parameters to describe microbial growth included lag phase duration, maximum growth rate in exponential phase, area under the growth curve and maximum value reached. To minimize micro-local leverages, the growth parameters were normalized analogously as described previously for the endpoint phenotyping. The growth parameters were first normalized to the dispersed grid control strains. Test plate (supplemented with NaCl) effect sizes were further normalized to the corresponding control values on YPD medium. In the case of isogenic allele swapping, relative growth parameters of the derived strains were finally normalized to the corresponding wild-type isogenic source strain values. Measurements of individual strains and their derivatives consisted of 6 replicates, for which the analysis was performed using custom R scripts. A two-tailed Student's t-test was used to compare different conditions; in comparisons to the reference, this value was set to 1.

### Genomic DNA extraction for short-read sequencing

Yeast genomic DNA for short-read whole-genome sequencing (WGS) was extracted by MasterPure Yeast DNA Purification Kit (#MPY80200, LGC, USA) according to the manufacturer's instructions. The pools of *S. cerevisiae* segregants were grown in spore selection medium either supplemented with the indicated NaCl concentration (extreme population) or without salt (reference population). After the harvest, *S. cerevisiae* cells were chemically lysed and proteins precipitated. The nucleic acids were precipitated from isopropanol and resuspended in TE buffer. Finally, RNAs were digested by RNase A (#740397, Macherey-Nagel, Germany) and genomic DNA was purified by a new protein and isopropanol precipitation steps. Thus extracted genomic DNA was degraded into medium-size (∼20 kb) fragments and was therefore suitable for short-read sequencing applications.

### WGS and bulk segregant analysis

Genomic DNA (prepared as described in the “Genomic DNA extraction for short-read sequencing” section) was sequenced on the short-read DNB-SEQ PE150 platform by BGI, Hong Kong, China. Fastq demultiplexed output files of PE150 whole genome sequencing were analyzed by publicly available tools and custom-made scripts (Pačnik et al. 2021). First, the reads within demultiplexed files were trimmed off of the adapters by bbduk. The trimmed reads were aligned to the reference sequence by bwa (Li and Durbin 2009), where the aligned reads were indexed and sorted by samtools (Li et al. 2009). Duplicates were marked by picard (Broad Institute 2019). Finally, the alternative variants were extracted by the HaplotypeCaller tool of the gatk 4.6 (Van der Auwera et al. 2013) and written into a .vcf file. For this and beforehand alignment purposes a reference *S. cerevisiae* S288C genome sequence (Cherry et al. 2012) was employed. The extracted variants were divided as SNV or indel type by gatk. Variants were further filtered for their probability (sequencing depth >100) and quality (quality by depth <2.0, rank sum test for mapping quality <−5.0) by the VariantFiltration tool of the gatk.

Reference genomes of the BY and CEN.PK parental strains were reconstructed by the picard-generated sequence dictionary according to the acquired variants. The pipeline was repeated for single genome analysis of individuals and for bulk segregant analysis of segregant pools. The data were extracted by the VariantsToTable function of the gatk tool and finally visualized by custom R scripts. For the single genome analysis of segregants with the most extreme trait value in each generation, we created a chromosome map with indicated source of the variants. For the bulk segregant analysis, we created a plot expressing a proportion of the variant source in test (i.e. supplemented with NaCl) and reference population (i.e. without salt), plotted against the genomic position of each detected variant.

### QTL identification

QTL mapping was performed by the R package QTLseqr (Mansfeld and Grumet 2018). It accommodates QTL-seq (Takagi et al. 2013) and G′ (Magwene et al. 2011) approaches which perform simulations and G statistics, respectively, to statistically identify and assess the QTLs. For this analysis, variants within two pools of segregants, grown in reference conditions without salt and extreme conditions supplemented with high NaCl salt concentration, were first extracted by HaplotypeCaller tool of the gatk 4.6 (Van der Auwera et al. 2013) to produce .gvcf files. Following this per-sample variant calling, a joint genotyping was performed across both samples to generate a single .vcf file by CombineGVCFs tool. Although the QTLseqr provides tools for variants filtration, we applied the same procedure and parameters with gatk's VariantFiltration tool as described for individual samples analyses. The filtered SNV data were transformed into a table with information on position and sequencing depth of reference (originating from the BY strain) and alternative variants (originating from the CEN.PK strain) in reference growth conditions (i.e. without salt) and extreme conditions (i.e. supplemented with high NaCl salt concentration) by VariantsToTable tool of gatk. This table was finally imported to the QTLseqr that estimates QTL probability based on SNV occurrence in reference and extreme conditions, compared to overall variant distribution. Applying the tricube smoothed G statistic (G′), *P*-values for QTL discovery are estimated for each SNV using non-parametric estimation of the null distribution of G′ (Magwene et al. 2011). Finally, −log_10_(*P*) values for the QTL discovery were plotted against genome coordinates in R to show areas of potential causative variants.

## Results

### Iterative backcrossing and high-throughput endpoint phenotyping

**Fig. 1.**
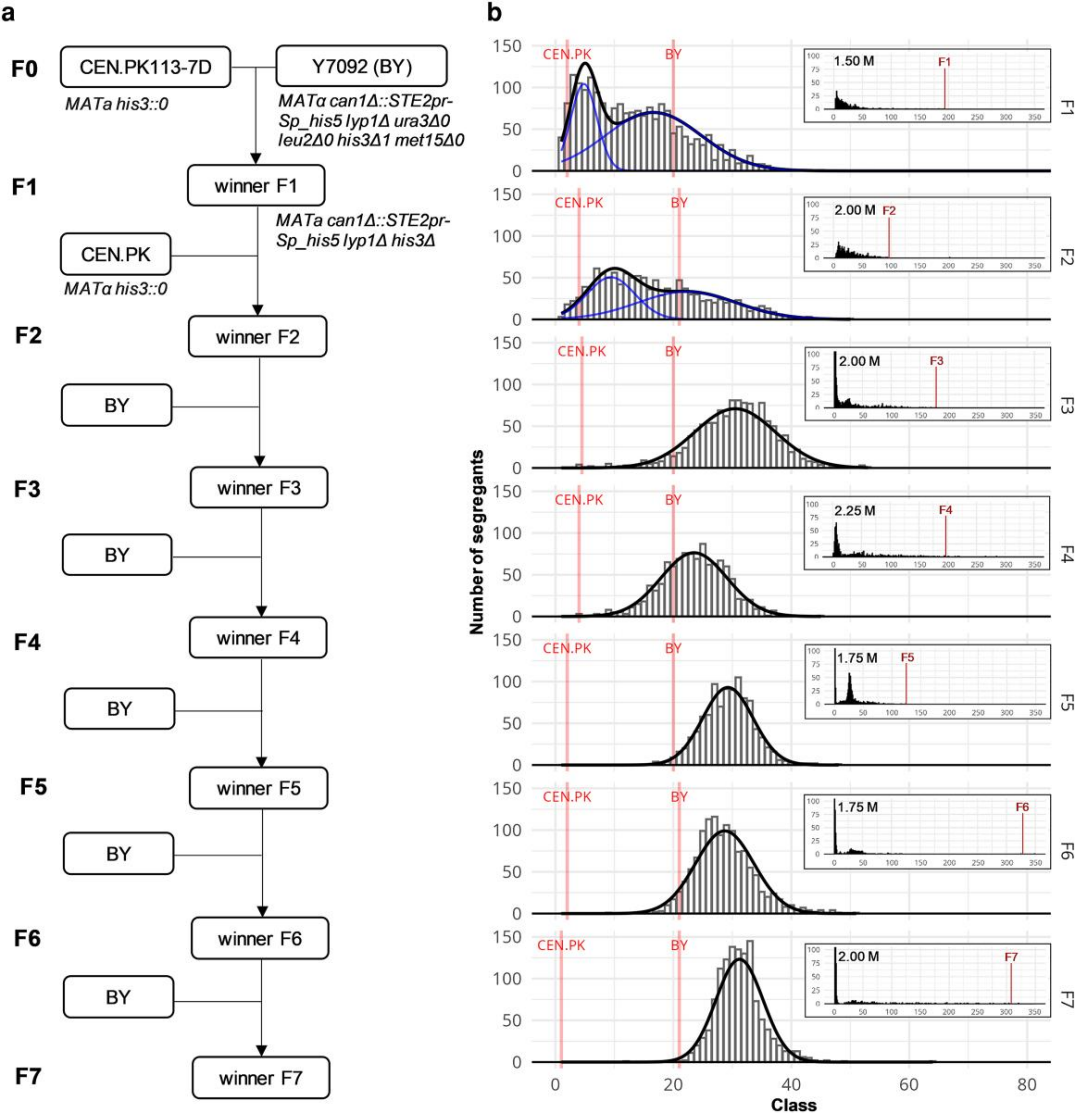
Iterative crossing scheme and the corresponding NaCl salt tolerance trait distribution. a) Iterative crossing, sporulation and selection plan of the experiment. In the first round, the selected laboratory parental strains with the indicated genotypes were crossed. The heterozygous diploid zygote (not indicated) was sporulated, the generated haploid offspring were phenotyped and the individual with the highest NaCl salt tolerance was selected (as presented in panel b). In the second step, the segregant with the most extreme trait value of the F1 generation was crossed with the CEN.PK parental strain to generate F2 segregants. In the following five iteration steps, the segregant with the most extreme trait value of each step was backcrossed with the BY parental strain. b) The histograms (gray) show the distribution of NaCl tolerance of haploid segregants across seven generations (F1–F7) of crossing, following the experimental plan (panel a). In the F1 generation, 2,156 segregants were analyzed, whereas in the subsequent generations the analyzed number was 1,232. The relative NaCl salt tolerance values, as calculated by the normalization to the reference colony size and to control plate without NaCl, were distributed into classes (*x*-axis) of size 0.05. Values of the parental strains are shown in red, where the reference BY parental strain falls into the 20th class by definition (i.e. its relative NaCl salt tolerance is 1). Gaussian mixture models were fitted to the experimental frequency distributions, with the resulting densities shown as black line-plots. The number of modes was inferred using the Bayesian Information Criterion (BIC), and bimodal distributions are highlighted with blue line-plots. In the presented case, colonies were grown for 3 d on YPD agar plates supplemented with 0.75 M NaCl, i.e., the conditions that still permitted growth of both parental strains. The segregants with the most extreme trait values in each generation of crossing were selected at higher NaCl concentrations, as shown in the inset distributions and marked in red. These selected segregants, exhibiting the most extreme phenotypes at the indicated NaCl concentrations, also displayed stable phenotypes at other high salt concentrations and ranked among the most salt-tolerant strains.

Two *S. cerevisiae* strains—BY and CEN.PK—with distinctive NaCl salt tolerance characteristics were chosen for the iterative crossing, haploid segregants production and QTL mapping. The strains were crossed according to a plan (Fig. 1a) where the most salt tolerant strain, i.e. the segregant with the most extreme (transgressive) trait value, of each generation was backcrossed with one of its parental strains. To generate F1 offspring, Y7092 (a BY derivative) and CEN.PK strains were crossed, heterozygous diploid sporulated and haploid segregants selected. Their NaCl salt tolerance was monitored daily by high-throughput endpoint phenotyping on solid YPD agar medium supplemented with NaCl in concentrations from 0.25 M to 2.50 M in increment of 0.25 M for four consecutive days with the representative results shown on Fig. 1b. To generate F2 offspring, a segregant with the most transgressive phenotype of the F1 generation was selected and crossed with the CEN.PK parental strain to retain causative variants originating from the less tolerant parental strain with higher probability. In the following five generations, the most salt tolerant segregant was backcrossed with its more salt tolerant BY parental strain to identify transgressive alleles (Fig. 1). In the last generation–theoretically, in neutral conditions and as expected by chance–only 2.34% of the opposite genetic variation would remain in the genome.

BY is a salt tolerant reference strain that exhibited an adequate growth rate in normal conditions (generation time of 1.5 h in YPD medium) and endured NaCl salt concentration up to 1.50 M. In our experiments, the industrial salt sensitive CEN.PK strain grew approximately 30% faster in normal conditions, yet its growth was nearly abolished at NaCl concentrations above 0.75 M. Distributions of the trait values indicated relatively high NaCl salt tolerance of the segregants which was detected at concentrations up to 2.25 M. The segregants therefore exhibited a highly transgressive phenotype with the majority of the population expressing more extreme trait value than the more salt tolerant parental strain. The trait value distributions were bimodal in the first two generations after crossing with the less salt tolerant CEN.PK parental strain and became unimodal in the subsequent generations when crossed with the BY parental strain (Fig. 1b). A significant increase in the population mean trait value was observed in the F3 generation. However, further efforts to improve NaCl salt tolerance did not significantly increase either the tolerance of the most extreme segregants or the population mean, but the variance was reduced.

### Whole-genome sequencing and QTL mapping

**Fig. 2.**
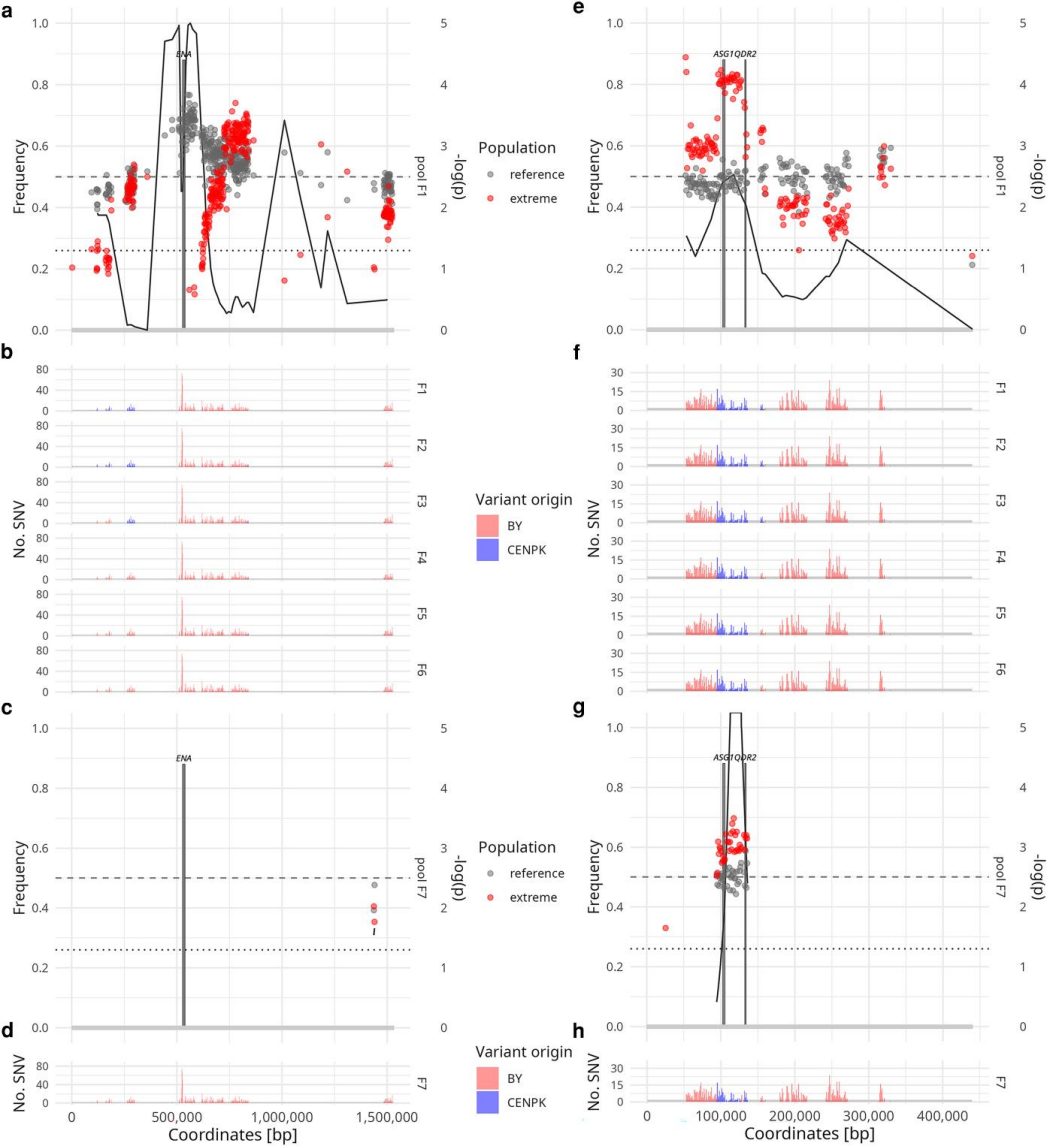
Quantitative trait loci (QTL) mapping. Analysis of variants on (a–d) chrIV and (e–h) chrIX revealed QTL areas that expressed the strongest impact on the trait's variability and were thus most likely causative. In the pool variant frequency plots (a, c; e, g), the frequencies of non-reference variants (sourced from the CEN.PK parental strain) are shown as median values within 1 kbp intervals, plotted against the genomic position (in reference coordinates) of each variant. For the extreme pool (supplemented with 1.50 M NaCl), non-reference variants are indicated in red, whereas for the reference pool (i.e. without NaCl) they are indicated in gray. Gray dashed line at frequency of 0.50 indicates variants with a neutral effect in the biparental population. Secondary y-axis on pool variant frequency plots (a, c; e, g) corresponds to the −log_10_(*P*) value of QTL discovery significance, calculated in interval of size 20,000 bp. Black dotted line at 1.30 corresponds to the *P*-value of 0.05, a significance threshold for QTL discovery. On panel a, a sharp drop in *P*-value is likely caused by low resolution of similar variants of *ENA* locus and estimation interval size. Variant source within individual segregants with the most extreme trait value (b, d; f, h) is presented as histograms of numbers of detected SNVs within 1 kbp at the genome position (coordination axis). Non-diverging sites are presented as gaps on the genome coordinate. Data on variant frequencies within segregant pools and variant composition of individual segregants with the most extreme trait value are shown in a consecutive experimental order (Fig. 1a). Areas of potential causative genes are marked.

Genomes of the parental strains and the segregants with the most transgressive trait value of each generation were sequenced (Fig. 2, Supplementary Fig. 1 in Supplementary File S1). A total of 21,100 SNVs were detected in the CEN.PK parental genome compared to the BY reference, slightly below the previously reported number of 21,899 (Nijkamp et al. 2012), corresponding to a heterozygosity level of 0.002. Additionally, pools of F1 and F7 segregants were sequenced in bulk where one half of the sample (∼10^4^ segregants) was grown in reference conditions lacking NaCl and the other half was in parallel grown in extreme conditions under high NaCl selection concentration (i.e. 1.50 M) such that ∼1% of the initial segregants survived. For the bulk segregant analysis, variant source and its sequencing depth were extracted at each diverging site. The analysis identified a total number of 22 potential causative loci (Supplementary Table 8 in Supplementary File S1) by highly altered variant frequencies from the equilibrium (i.e. 0.50) within the high NaCl concentration pool of segregants compared to its levels in reference conditions (Supplementary Fig. 1 in Supplementary File S1). Variant composition of these potential causative loci within individual segregants with the most extreme trait value of each generation was monitored. Among the predicted QTLs, candidates with (i) the highest discovery significance, (ii) persistence throughout the backcrossing steps, and (iii) a reduction of the size of the loci were observed for two QTLs, one on chrIV (152 kbp between coordinates 503,000 and 655,000) and the other on chrIX (37 kbp between coordinates 97,000 and 134,000) (Fig. 2).

The QTL on chrIV had a high predicted impact on NaCl salt tolerance (Fig. 2a), as in the reference conditions the frequency of the CEN.PK variants was up to 0.75, whereas in the extreme salinity conditions the frequency dropped to 0.10 within the F1 pool (−log_10_(*P*) = 5). Throughout the following backcrossing steps, the CEN.PK variants were completely lost from the population (Fig. 2, b–d). The proposed QTL on chrIX shrank through the iterative backcrossing steps (Fig. 2, f and h), dismissing the accompanying non-causative variants. The frequency of CEN.PK and BY variants within the locus remained equal in reference conditions, whereas in the F1 and F7 extreme salinity pools the frequencies of the CEN.PK variants were elevated to 0.80 (−log_10_(*P*) = 2.5) and 0.65 (−log_10_(*P*) > 5), respectively (Fig. 2, e and g).

### Candidate gene evaluation

Within the QTL on chrIV, the most promising hit was the *ENA* locus that is directly involved in osmotic and NaCl stress tolerance (Tamás and Hohmann 2007; Treusch et al. 2015). The *ENA* locus of the more salt tolerant BY strain includes genes *ENA1*, *ENA2* and *ENA5* (i.e. *ENA^BY^*), whereas the same locus in the genome of the salt sensitive CEN.PK strain includes a single gene *ENA6* (i.e. *ENA^CEN.PK^*). The proposed QTL on chrIX consisted of 14 genes (Supplementary Table 8 in Supplementary File S1), of which we considered *ASG1* and *QDR2*, as they are related to the osmotic stress response function (Vargas et al. 2007; Ríos et al. 2013; Xu et al. 2014), *KGD1* and *AYR1*, as they were the most outstanding hits in the F1 pool, and *MET18* and *RRT14*, as they were the most outstanding hits in the F7 pool of segregants (Fig. 2, e and g).

**Fig. 3.**
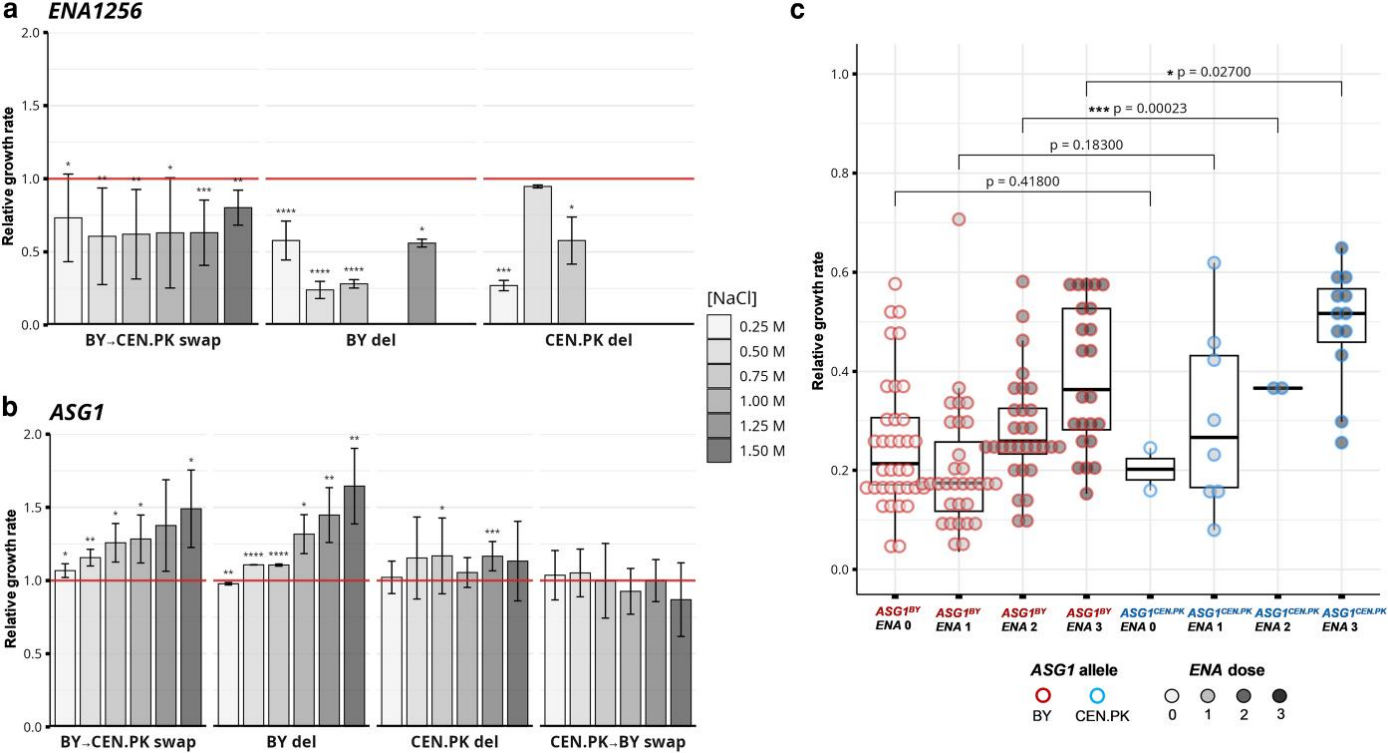
Evaluation of quantitative trait genes (QTGs) by allele swapping and their genotype-to-phenotype association. a, b) Effect on maximum growth rate as a NaCl salt tolerance phenotype by manipulation of QTGs on (a) *ENA* locus of chrIV and (b) *ASG1* gene of chrIX. The histograms show relative values of maximum growth rate (y-axis) of isogenic derivatives with respect to the source wild-type strains. The strain derivatives were grouped (x-axis) by the parental allele deletion (del) and the direction of the allele swap where BY allele was swapped with CEN.PK allele (BY → CEN.PK swap) or *vice versa*, i.e. CEN.PK allele was swapped with BY allele (CEN.PK → BY swap). The continuous growth assessments were performed within increasing NaCl concentrations, as indicated. The error bars represent one standard deviation where BY parental allele group consisted of 12 different strains for *ENA* locus and 2 for *ASG1* gene, and CEN.PK parental allele group consisted of 4 different strains for *ENA* locus and 14 for *ASG1* gene, and each treatment included 6 biological replicates. Red horizontal line at the relative value of 1.0 corresponds to the source isogenic wild-type strain value and denotes a gene manipulation effect on the maximum growth rate. Statistical significance was determined using a two-sided t-test against the reference value of 1 that corresponds to the relative value of the source isogenic strain. Significance is indicated as follows: *P* < 0.05 (*), *P* < 0.01 (**), *P* < 0.001 (***), and *P* < 0.0001 (****). Additional corresponding results are shown on Supplementary Fig. 2 in Supplementary File S1. c) Collection-wide genotype-to-phenotype association of *ASG1* alleles and *ENA* dose for NaCl tolerance. Phenotype data on relative growth in 1.0 M NaCl for combinations of *ASG1* allele and *ENA* dose were extracted from The 1002 Yeast Genomes Project (Peter et al. 2018) an pooled with our data. Boxplots show the median (center line), interquartile range (box), and minimum/maximum values excluding outliers (whiskers). Individual points represent biological replicates. BY and CEN.PK alleles of the *ASG1* gene are indicated by red and blue dot borders, respectively, while *ENA* gene dose is represented by varying shades of gray in the dot fill. Statistical significance was determined using a two-sided t-test comparing the two groups.

The candidate genes within QTLs on chrIV and chrIX were firstly deleted and then swapped for their alternative alleles by scarless CRISPR-Cas9 procedure. The allele deletion and swap experiments were conducted on 16 different *S. cerevisiae* genotyped strains from this study. The strain panel included parental strains BY, Y7092 and CEN.PK from a yeast collection, as well as 13 segregants from the BY × CEN.PK crosses, 7 of which represented individuals with the most extreme trait values from each generation of crossing. The variant effect, i.e. relative growth, was quantitatively assessed within the generated isogenic strain backgrounds by continuous growth rate measurement on solid YPD agar plates supplemented with NaCl in concentrations ranging from 0.25 M to 1.50 M. We determined the isogenic variant effect on quantitative growth parameters lag phase duration, maximum growth rate in exponential phase, total area under the curve and maximum value of the growth curve (Fig. 3, a and b, Supplementary Fig. 2 in Supplementary File S1).

A complete deletion of either variant of the *ENA* locus decreased maximum growth rate already at lower NaCl concentrations for 40–75% on average, compared to the source wild-type strains, while at NaCl concentrations above 0.75 M the growth of deletion mutants was completely abolished (Fig. 3a). At the lower NaCl concentrations from 0.25 M to 0.75 M the swap of *ENA^BY^* locus with *ENA^CEN.PK^* locus decreased maximum growth for 25–40% on average, compared to the source wild-type strains. We were not able to perform the swap of the *ENA^CEN.PK^* locus with the *ENA^BY^* locus due to a high degree of sequence similarity between the alleles (97%) and because of the locus length (11 kbp). As in the case of the maximum growth rate, deletion of the *ENA* locus in either of the strain backgrounds and the swap with the *ENA^CEN.PK^* variant prolonged the lag phase duration and decreased the reached maximum value of the growth curve and the area underneath it (Supplementary Fig. 2g in Supplementary File S1). Additionally, we observed a considerable positive correlation between the gene dose, i.e. the number of *ENA* gene copies, and NaCl salt tolerance in our laboratory biparental experiment (Supplementary Fig. 3 in Supplementary File S1). This correlation effect was not dependent on NaCl concentration and was evident already at 0.25 M NaCl, whereby higher concentrations proportionally reduced the growth rate. The swap of the three-gene *ENA^BY^* locus with the one-gene *ENA^CEN.PK^* locus partially restored NaCl salt tolerance, as it increased the tolerance for 40–60% on average at lower NaCl concentrations from 0.25 M to 0.75 M compared to the strain with the complete deletion of the *ENA* locus. The maximum growth rate of the swapped derivatives was still 25–40% lower than the growth rate of the corresponding strains with the three-gene locus.

Due to the observed allele swap effect, we confirmed the Activator of Stress Genes 1 (*ASG1*) gene to be the causative QTG on the QTL of chrIX (Fig. 3b) with the variant from the BY parental strain (i.e. *ASG1^BY^*) negatively affecting NaCl salt tolerance compared to the CEN.PK parental strain variant (i.e. *ASG1^CEN.PK^*). With the increase of supplemented NaCl concentration from 0.25 M to 1.50 M, the maximum relative growth rates due to *ASG1^BY^* deletion gradually increased from 0.98 ± 0.08 to 1.65 ± 0.26 compared to the source wild-type strain, whereby errors denote standard deviation. Similarly, swap of the *ASG1^BY^* allele with the *ASG1^CEN.PK^* caused a gradual increase of the maximum growth rate from 1.07 ± 0.05 to 1.49 ± 0.26 compared to the source wild-type strain. Additionally, concentration-dependent effect of the *ASG1* gene manipulation was observed for the reached maximum value of the growth curve and area underneath it whereby both, deletion of the *ASG1^BY^* allele or its swap with the *ASG1^CEN.PK^* allele gradually increased the relative values of growth parameters (Supplementary Fig. 2a in Supplementary File S1). Conversely, swap of the *ASG1^CEN.PK^* allele with the *ASG1^BY^* allele as well as deletion of the *ASG1^CEN.PK^* allele did not significantly affect the relative growth rate or other growth parameters (Supplementary Fig. 2a in Supplementary File S1). *ASG1* gene manipulation, however, did not affect the lag phase duration significantly.

To explore the general *ASG1*-allele-dependent effect on NaCl salt tolerance, we conducted genotype-to-phenotype association analysis across the 1,011 *S. cerevisiae* isolate collection (Peter et al. 2018). In addition to the generated strain derivatives, 127 haploid isolates from the collection were categorized based on carrying either an *ASG1^B^*^Y^- or *ASG1^CEN.PK^*-like allele. The categorization was driven by the length of the Asn homo-repeats, a presumed diversification characteristic in the primary protein structure, with Asg1^BY^ variant containing a tract of 17 consecutive Asn residues, compared to 24 in Asg1^CEN.PK^ (see Discussion and Supplementary Fig. 4 in Supplementary File S1 for more detail). The longer Asn homo-repeat motif (≥Asn_20_) indicated the presence of *ASG1^CEN.PK^*-like allele, and a shorter one (<Asn_20_) indicated the presence of *ASG1^BY^*-like allele. Given that *ENA* gene dose appeared to be the primary determinant of NaCl salt tolerance (as shown on Supplementary Fig. 3 in Supplementary File S1), a second stratification was applied based on the *ENA* gene copy number (0, 1, 2, or 3 copies). The *ENA* gene doses of 1 and 3 originated from the CEN.PK and BY parental strains, respectively, whereas a dose of 0 reflected a complete deletion of the *ENA* locus, and a dose of 2 resulted from natural variation observed in the isolate collection. Relative growth rates in 1 M NaCl compared to reference media conditions without salt were extracted (Peter et al. 2018) and presented according to the combinations of *ASG1* allele and *ENA* dose (Fig. 3c). Although the *ASG1^CEN.PK^*-like allele was relatively rare in the population, significant differences were observed between strains with either *ASG1^B^*^Y^ or *ASG1^CEN.PK^* allele at *ENA* doses of 2 and 3 copies. On average, relative growth rates of strains with the *ASG1^CEN.PK^* allele were 30% ± 12% and 27% ± 12% higher as those with *ASG1^B^*^Y^ at 2 and 3 copies of the *ENA* genes, respectively (errors denote standard deviation).

## Discussion

In the pursuit of QTLs for NaCl salt tolerance, the distribution of the trait in the population of segregants from BY and CEN.PK parental strains cross was monitored through seven generations of iterative crossing. Trait distribution in the first two generations of segregants, the offspring of crosses between CEN.PK parental strain with BY parental strain and segregant of F1 with the most extreme phenotype, was bimodal and thus primarily affected by a single high effect gene. The subsequent crosses of BY parental strain and segregants with the most extreme trait values resulted in populations with presence of only the superior variant of the *ENA* locus and showed a unimodal distribution, indicating a polygenic nature of the trait within the salt tolerant pool of strains. When the strongest-impact variant was fixed, additional variants influenced the distribution, reflecting the background-dependent inheritance pattern (Hou et al. 2016, 2018) and reinforcing the premise that a monogenic trait could be genetically more complex than initially described. From the F3 generation onward, the majority of the population exhibited a phenotype more extreme than that of the more NaCl salt tolerant BY parental strain used for the crosses. Additionally, trait values of the most salt tolerant segregants increased most notably in the F1 and F2 generations of crossing, following the introduction of transgressive alleles from the less salt-tolerant CEN.PK parental strain. Subsequent iterations of backcrossing with the more salt tolerant parental strain yielded smaller improvements in maximum NaCl tolerance values. Beyond the F3 generation, population-level phenotypic progression was negligible, although the distribution around the mean narrowed as a result of backcrossings. QTLs with positive effects were fixed, with those evaluated here on chrIV and chrIX having the strongest impact.

Within the *ENA* locus on chrIV, a sharp drop in the frequency of CEN.PK variants in the F1 pool of the segregants under high salinity conditions with a corresponding QTL discovery *P*-value of 10^−^⁵ indicated the presence of a major causative locus. The role of the *ENA* locus as a key determinant of NaCl salt tolerance was further supported by population distribution analysis. When both variants of the *ENA* locus were present in the population, the trait distribution was bimodal, with the mean values close to the BY and CEN.PK parental values, whereas presence of only one of the variants resulted in a monomodal distribution. The observed strong effects and molecular role of the *ENA* locus have already been associated with osmotic stress tolerance (Tamás and Hohmann 2007; Daran-Lapujade et al. 2009; Anderson et al. 2010; Treusch et al. 2015). However, although identification and evaluation of the *ENA* locus are not entirely novel, its inclusion in the analysis was essential, as its major contribution to NaCl salt tolerance would otherwise mask the effects of other loci.

The second-impact QTL, identified with a discovery *P*-value of 10^−2.5^ and 10^−5^ in the F1 and F7 generation pools, respectively, was located on chrIX. This QTL not only reflected pronounced differences in variant frequencies in F1 and F7 generation pools, but was also narrowed through the crossing process, eliminating non-causative variants. Within this QTL, we confirmed Activator of Stress Genes 1 (*ASG1*) as the causative gene. Deletion of the *ASG1^BY^* allele or swap of the *ASG1^BY^* allele with *ASG1^CEN.PK^* demonstrated a similar positive effect on NaCl salt tolerance in a concentration-dependent manner, whereas *ASG1^CEN.PK^* allele deletion or swap of the *ASG1^CEN.PK^* allele with the *ASG1^BY^* did not affect NaCl tolerance. Integrating all available data, we conclude that the *ASG1^BY^* allele actively decreased NaCl salt tolerance, whereas we found no evidence that the transgressive *ASG1^CEN.PK^* allele improved it. Deletion of *ASG1^CEN.PK^* allele did not affect the NaCl salt tolerance phenotype and Asg1^CEN.PK^ variant was therefore presumed to have a neutral effect, or, possibly, to be inactive.

Our hypothesis on the *ASG1* gene allele-dependency was confirmed through a specie-wide genotype-to-phenotype association analysis. To control for the strong influence of the *ENA* locus, the *ASG1*-allele effect was restricted to comparisons within haploid strains with the same *ENA* dose. A significant advantage in NaCl salt tolerance was observed for strains carrying the *ASG1^CEN.PK^* allele compared to those with the *ASG1^BY^* allele at 1 M NaCl in strains with *ENA* copy numbers of 2 and 3. No significant effect was detected at copy numbers of 0 or 1, presumably due to insufficient growth of strains with low *ENA* dose under high-salinity conditions. These data, however, exhibited greater variability than the allele swapping experiment, likely due to background-dependent effects arising from the normalization method as the growth rates were normalized only to reference conditions (i.e. YPD without salt) and not to isogenic derivatives.

On a molecular level, Activator of Stress Genes 1 (Asg1) is a proposed transcriptional regulator and is presumably involved in lipid metabolism and stress response (Xu et al. 2014; Jansuriyakul et al. 2016). It has been reported to activate stress response genes against a wide array of stressors, such as autolysis (Xu et al. 2014) and oxidative stress that is directly related to β-oxidation (Jansuriyakul et al. 2016), while also being involved in response to osmotic stress (Fierling et al. 2024). The roles of Asg1 remain ambiguous as the reference yeast strain (BY) with deleted *ASG1* gene is unresponsive to fatty acid utilization, unaffected by oxidative stress (Jansuriyakul et al. 2016) and resistant to osmotic stress (Fierling et al. 2024). Our results on NaCl salt tolerance are largely aligned with the reported observations that Asg1 protein variant of the reference background (Asg1^BY^) acts differently from other variants. We showed that Asg1^BY^ negatively affects NaCl tolerance with isogenic deletion mutant exhibiting higher tolerance. This is consistent with its identification in a reference BY strain gene set of 60 candidates in a systematic knock-out mutation collection high-throughput experiments for Li^+^ and Na^+^ ions sensitivity (Fierling et al. 2024). We attempted to clarify the observed contrasting effects of Asg1^BY^ and Asg1^CEN.PK^ variants on a functional level. Primary structures of the protein variants are 98.6% identical with the gap frequency representing most (0.9%) of the divergence. Structurally, while their functional Zn^2+^_2_-Cys_6_ fungal-type DNA-binding domains are identical, they could not be completely aligned in the predicted model (Jumper et al. 2021) due to differences in other areas (Supplementary Fig. 4 in Supplementary File S1). The positions at which the variants are most divergent were not structured, thus based on structural differences we could not predict the molecular mechanism of the Asg1 effect. Additionally, no significant results have been reported for NaCl-induced osmotic stress related *ASG1* transcription alteration in different laboratory and domesticated strains (Posas et al. 2000; Rep et al. 2000; O’Rourke and Herskowitz 2004; Hirasawa et al. 2006; Gat-Viks and Shamir 2007; Melamed et al. 2008), although the transcriptional response to high salinity in *S. cerevisiae* is overall well studied (Melamed et al. 2008). Whereas the core promotor regions of the variants are not identical, no regulation-dependent effect could have been observed in our study as we only swapped the coding regions. Translation rate of the variants may, however, be influenced by the codon usage. Global minimum score in optimal translation (Rodriguez et al. 2018) of the *ASG1* gene is reached within the area where Asg1^CEN.PK^ variant possesses an addition of 7 consecutive Asn residues (Supplementary Fig. 4 in Supplementary File S1). Within this region, the predicted translation of the *ASG1^CEN.PK^* is by 36% less optimal than *ASG1^BY^*, which may restrict protein expression and mitigate its negative effects on NaCl salt tolerance.

## Conclusions

It has been estimated that up to 40 causative variants impact response to chemical perturbations in *S. cerevisiae* (Ehrenreich et al. 2010, 2012; She and Jarosz 2018). Although the sole strong role of the *ENA* locus on NaCl salt tolerance indicates an almost monogenic nature of the trait (Anderson et al. 2010; Hou et al. 2016, 2018; Sirr et al. 2018), background-specific distributions suggest that the number of contributing factors is in fact higher (Hou et al. 2016, 2018). In our study, we proposed 22 potential QTLs that affect NaCl salt tolerance and arose from an inbred biparental population using a genome-wide QTL mapping approach. As an already marginal effect may be partly influenced by genetic interaction network (Fay 2013; Hou et al. 2018), we applied here an isogenic candidate allele-swap approach to quantitatively assess the contribution of potential causative genes. We concluded that Asg1 activity negatively affected NaCl salt tolerance and since Asg1 is presumed to function as a transcriptional regulator (Xu et al. 2014; Jansuriyakul et al. 2016), we hypothesized that it inhibits expression of stress-response genes that would otherwise act to neutralize the detrimental effects caused by NaCl. Since more QTGs likely reside within the remaining QTLs, we suggest that in order to identify additional causative genes, superior alleles of the major impact *ENA* and *ASG1* loci should be fixed in the parental strains so that alleles with smaller contribution would be unmasked (Fay 2013).

## Supplementary Material

jkaf254_Supplementary_Data

## Data Availability

The data on the whole-genome short-read sequencing underlying this article are available in GenBank Sequence Read Archive (SRA) repository. They can be accessed with BioProject accession number PRJNA1230855. The corresponding genotype data with extracted variants are available on FigShare (doi:10.6084/m9.figshare.29820095). End-point phenotyping data on population-dependent distributions are available on FigShare (doi:10.6084/m9.figshare.29820164). Continuous phenotyping data, including growth parameters, are available on FigShare (doi:10.6084/m9.figshare.29820191). Strains and plasmids generated in this study are available upon request. Previously published genotype and phenotype data from the 1,011 S. cerevisiae isolate collection (Peter et al. 2018) were used in this study and are accessible at http://1002genomes.u-strasbg.fr. Supplemental material available at G3 online.
