# Supplementary material for: Discovery and validation of ASG1 as a novel determinant of NaCl tolerance in the yeast Saccharomyces cerevisiae through iterative crossing
Source: G3 (Bethesda). 2025 Oct 28;16(1):jkaf254. doi: 10.1093/g3journal/jkaf254 (PMC12774596; doi:10.1093/g3journal/jkaf254)
Supplement: jkaf254_Supplementary_Data [file jkaf254_supplementary_data.pdf]

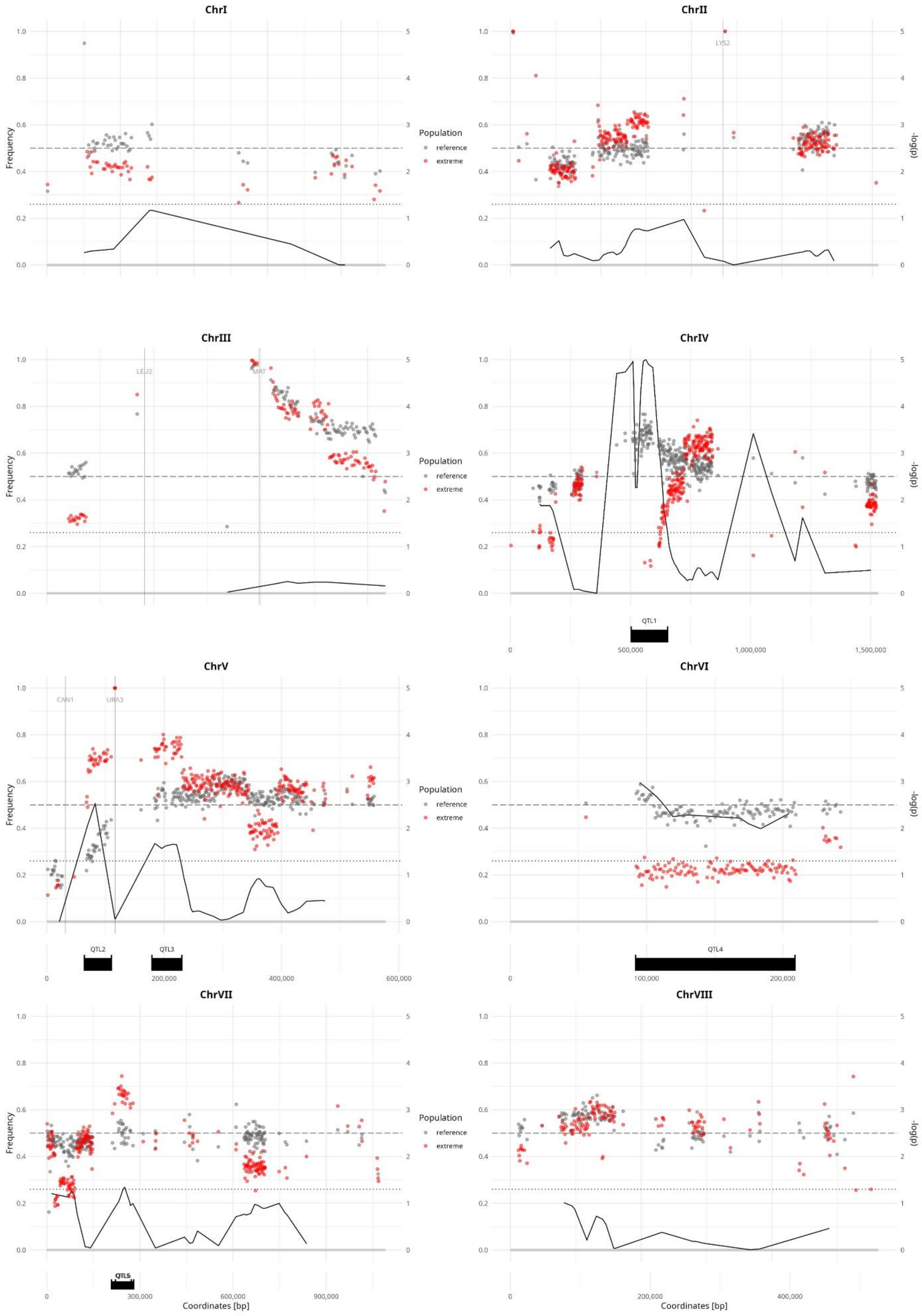
ChrI
ChrII
ChrIII
ChrIV
ChrV
ChrVI
ChrVII
ChrVIII
Frequency
-log(p)
Population
reference
extreme
LYS2
LEU2
MAT
CAN1
URA3
QTL1
QTL2
QTL3
QTL4
QTL5
Coordinates [bp]

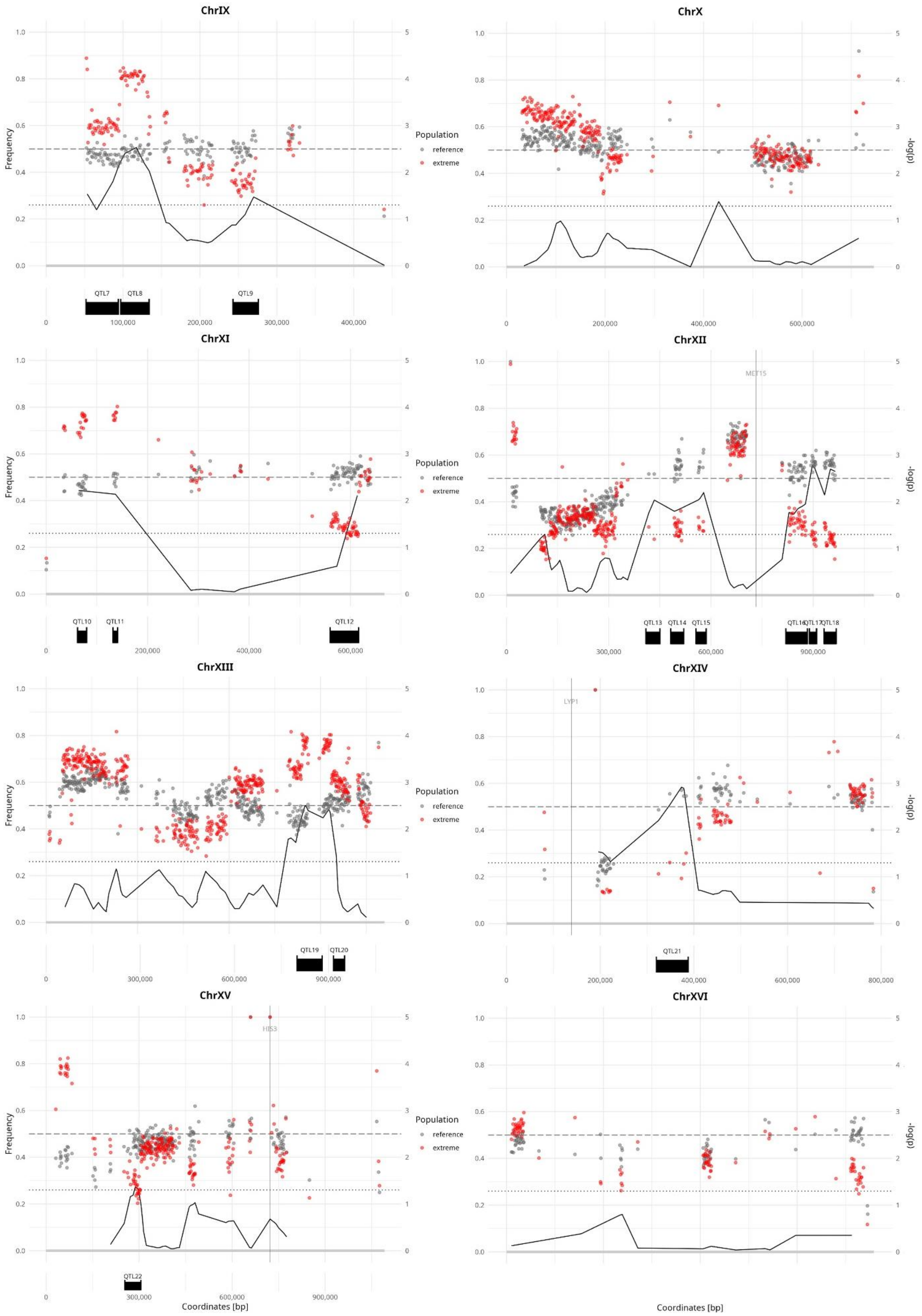
ChrIX
ChrX
ChrXI
ChrXII
ChrXIII
ChrXIV
ChrXV
ChrXVI
Frequency
-log(p)
Population
reference
extreme
QTL7
QTL8
QTL9
QTL10
QTL11
QTL12
QTL13
QTL14
QTL15
QTL16
QTL17
QTL18
QTL19
QTL20
QTL21
QTL22
MET15
LYP1
HIS3
Coordinates [bp]

Supplementary Figure 1: **Quantitative trait loci (QTL) mapping throughout the genome (extended Figure 2).** The most informative for causative loci examination were comparisons of extreme (high NaCl salt concentration) and reference (without supplemented salt) pools of segregants. Data for the F1 pool of segregants where population in extreme parallel was treated by 1.50 M NaCl are shown. At each diverging site, variant source and its sequencing depth were extracted. On the plots, the frequencies of non-reference variants (sourced from the CEN.PK parental strain) are shown on the primary y-axis as median values within 1 kbp intervals, plotted against the genomic position (in reference coordinates) of each variant. For the extreme pool, non-reference variants (sourced from the CEN.PK parental strain) are indicated in red, whereas for the reference pool they are indicated in grey. Grey dashed line at frequency of 0.50 indicates variants with a neutral effect in the biparental population. Secondary y-axis corresponds to the $-\log_{10}(p)$ values for QTL discovery that are presented by black lines. The values were calculated by tricube smoothed G statistic for each SNV using non-parametric estimation of the null distribution in interval of size 20,000 bp by the QTLseqr tool (Mansfeld and Grumet 2018). Black dotted line at 1.30 corresponds to the p-value of 0.05, a significance threshold for QTL discovery. Variant frequencies at all chromosomes are shown separately. Bellow the coordinates axis, areas of all detected potential 22 QTLs throughout the genome are indicated. Vertical bars indicate positions of marker genes used in the segregant selection process.

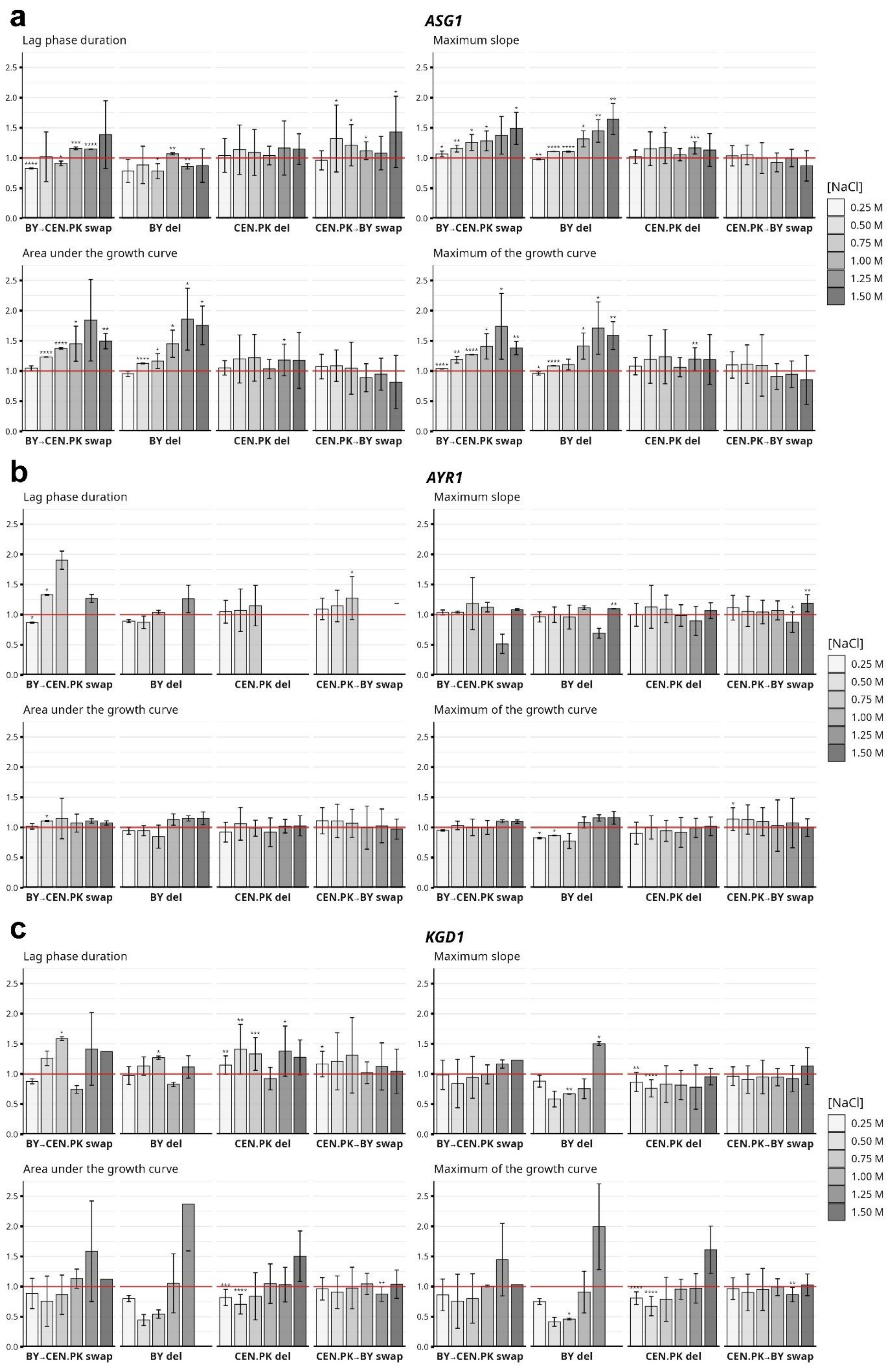
a
ASG1
Lag phase duration
Maximum slope
Area under the growth curve
Maximum of the growth curve
BY→CEN.PK swap
BY del
CEN.PK del
CEN.PK→BY swap
[NaCl]
0.25 M
0.50 M
0.75 M
1.00 M
1.25 M
1.50 M
b
AYR1
Lag phase duration
Maximum slope
Area under the growth curve
Maximum of the growth curve
c
KGD1
Lag phase duration
Maximum slope
Area under the growth curve
Maximum of the growth curve

d

MET18

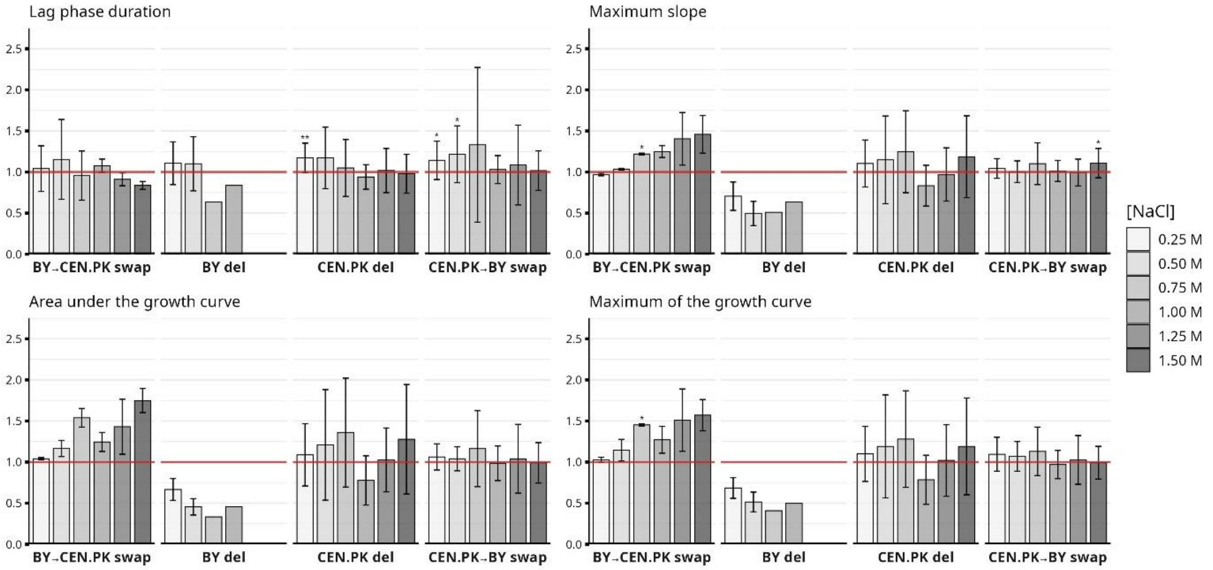

e

QDR2

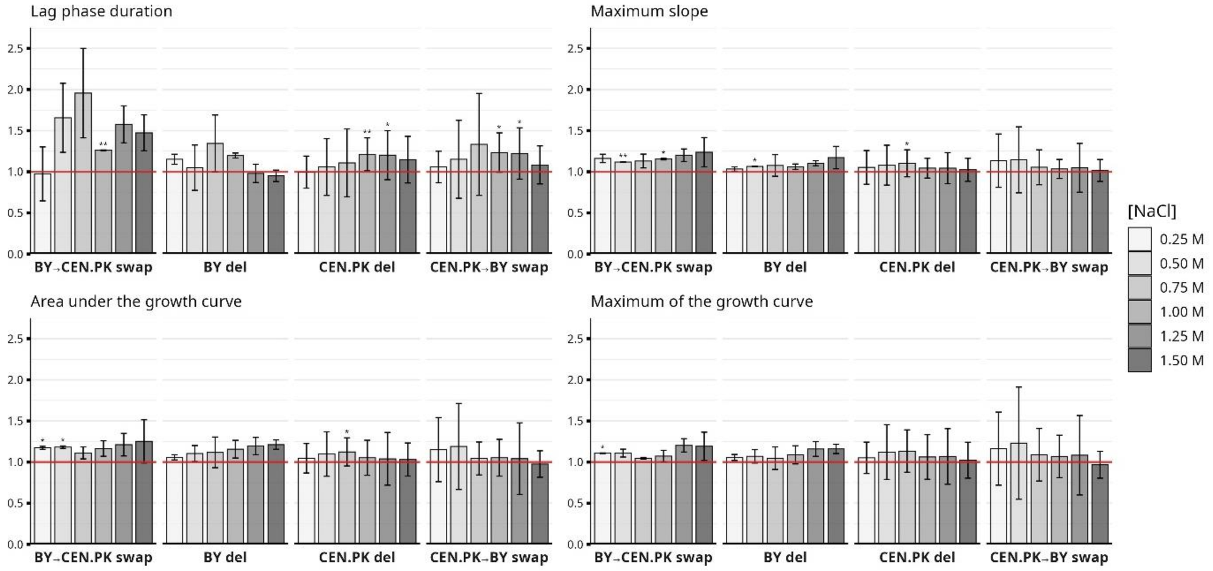

f

RRT14

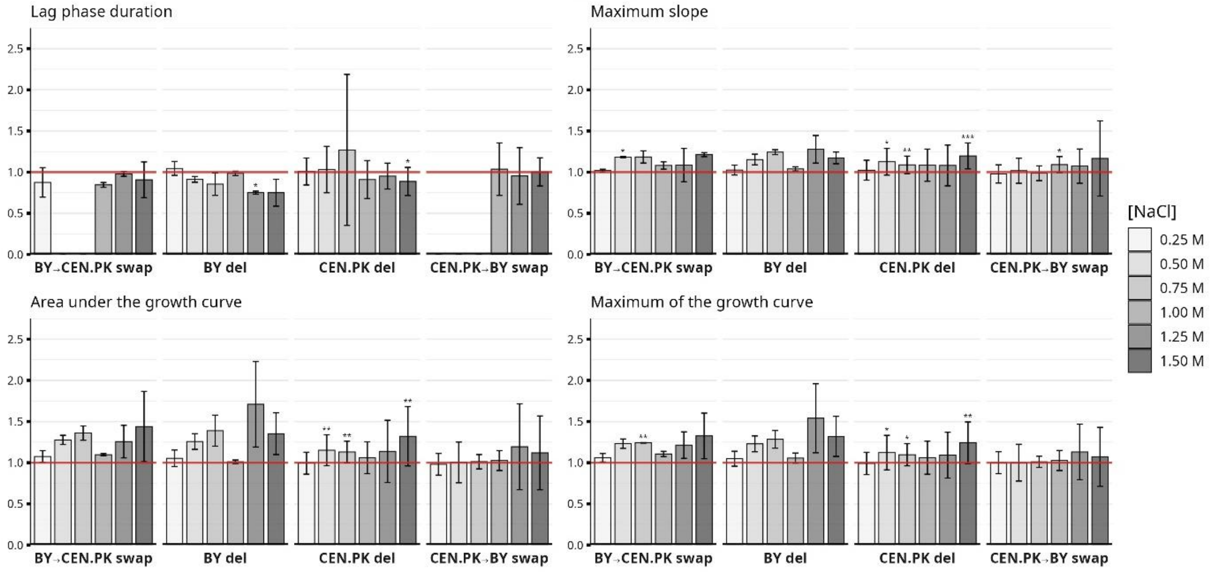

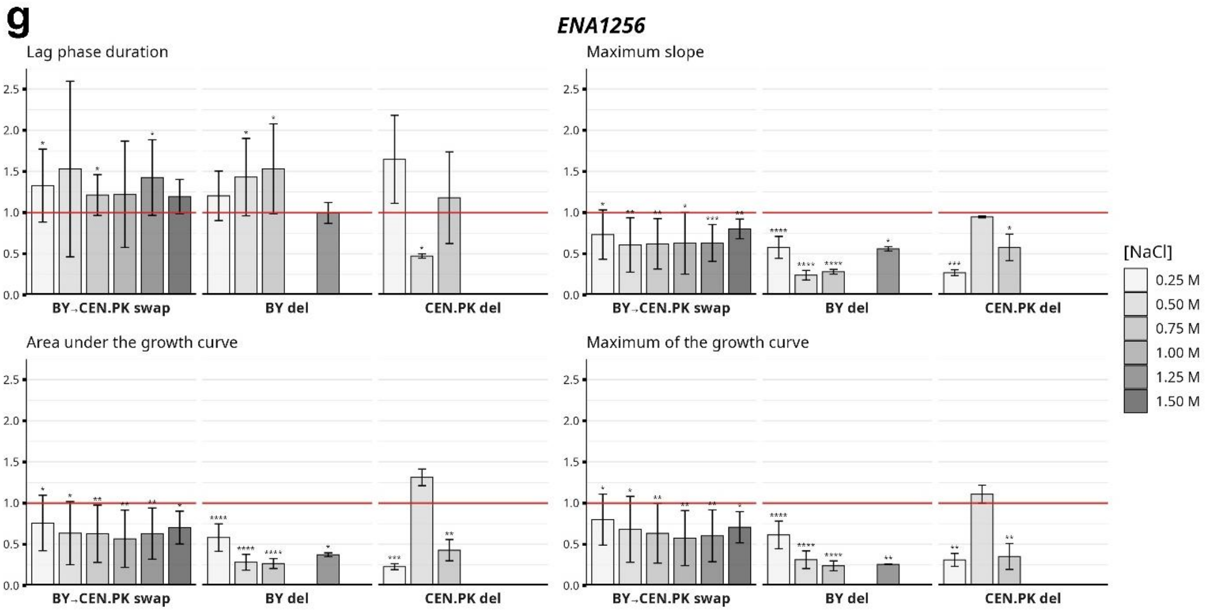

Supplementary Figure 2: **Quantitative trait loci (QTGs) evaluation by allele swap (extended Figure 4).** To test gene causality, the wild-type source allele was deleted and swapped for its alternative variant and the corresponding NaCl salt tolerance of all three isogenic derivatives was compared. On the chrIX, the proposed potentially causative genes were *ASG1* (**a**), *AYR1* (**b**), *KGD1* (**c**), *MET18* (**d**), *QDR2* (**e**) and *RRT14* (**f**), whereas on the chrIV the causative genes were from the *ENA* locus (**g**). The strain derivatives were grouped (x-axis) by the parental allele deletion (del) and direction of the allele swap where BY allele was swapped with CEN.PK allele (BY→CEN.PK swap) or *vice versa*, i.e. CEN.PK allele was swapped with BY allele (CEN.PK→BY swap). Growth of the source wild-type strains and derivatives within isogenic backgrounds was monitored on solid YPD media plates with the indicated NaCl concentration in the range from 0.25 M to 1.50 M with the increment of 0.25 M. Quantitative growth parameters were extracted and growth values relative to the wild-type strains calculated (y-axis). Presented parameters that describe the growth curve are i) lag phase duration, ii) maximum slope, iii) area under the curve and iv) maximum value of the growth curve. Error bars represent one standard deviation of uncertainty. To generalize the NaCl salt-induced growth effect, BY parental allele group consisted of 12 different strains for *ENA* locus and 2 for locus on chrIX and CEN.PK parental allele group consisted of 4 different strains for *ENA* locus and 14 for locus on chrIX and each treatment included 6 biological replicates. Red horizontal line at the relative value of 1.0 corresponds to the source wild-type strain value and denotes a gene manipulation effect on the growth parameters. Statistical significance was determined using a two-sided t-test against the reference value of 1 that corresponds to the relative value of the source isogenic strain. Significance is indicated as follows: $p < 0.05$ (*), $p < 0.01$ (**), $p < 0.001$ (***), and $p < 0.0001$ (****).

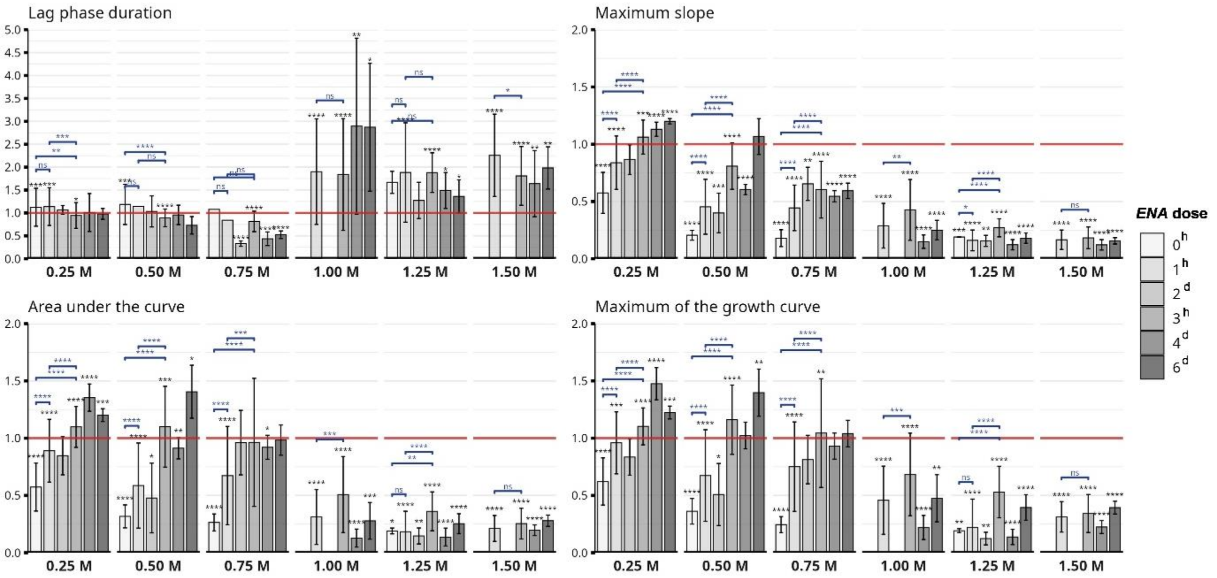

Supplementary Figure 3: ***ENA* gene dose dependent NaCl salt tolerance.** Salt tolerance is presented as a function of the *ENA* gene dose (shown with shades of grey), where doses of 0, 1, and 3 genes correspond to haploid strains (h) and doses of 2, 4, and 6 genes correspond to diploid form of strains (d). The trait was assessed from high-throughput growth curve measurements from which quantitative parameters – i) lag phase duration, ii) maximum slope, iii) area under the curve, and iv) maximum value of the growth curve – were extracted. On y-axes, relative NaCl salt tolerance is presented where growth parameters of the individual strains in test conditions were normalized to their corresponding growth in standard (without NaCl) conditions. *ENA* gene dose dependent effect was evaluated in NaCl concentrations from 0.25 M to 1.50 M, as indicated (x-axes). Red vertical line at 1.0 expresses no salt induced effect, as the strains would grow equally in saline and standard conditions. Dose of the **0** *ENA* genes was determined within all deletion mutants from the allele swap assay (16), dose of **1** *ENA6* gene within CEN.PK locus source strains (4) and allele swapped strains (12), dose of **2** doubled *ENA6* genes within CEN.PK diploid strain (1), dose of **3** *ENA1*, *ENA2* and *ENA5* genes within BY locus source strains (12), dose of **4** *ENA1*, *ENA2*, *ENA5* and *ENA6* genes within F1 (BY × CEN.PK) heterozygous diploid strain (1), and dose of **6** doubled *ENA1*, *ENA2*, *ENA5* genes within BY diploid strain (1). Each treatment included 6 biological replicates and empty bar-plots indicate absence of adequate growth. Error bars express one standard deviation of uncertainty. Statistical significance was determined using a two-sided t-test against the reference value of 1 that corresponds to the NaCl salt-induced effect threshold. For comparisons between groups (presented in dark blue) of haploid strains with 0, 1 and 3 *ENA* gene copies statistical significance was determined using a two-sided t-test. Significance is indicated as follows: $p < 0.05$ (*), $p < 0.01$ (**), $p < 0.001$ (***), and $p < 0.0001$ (****).

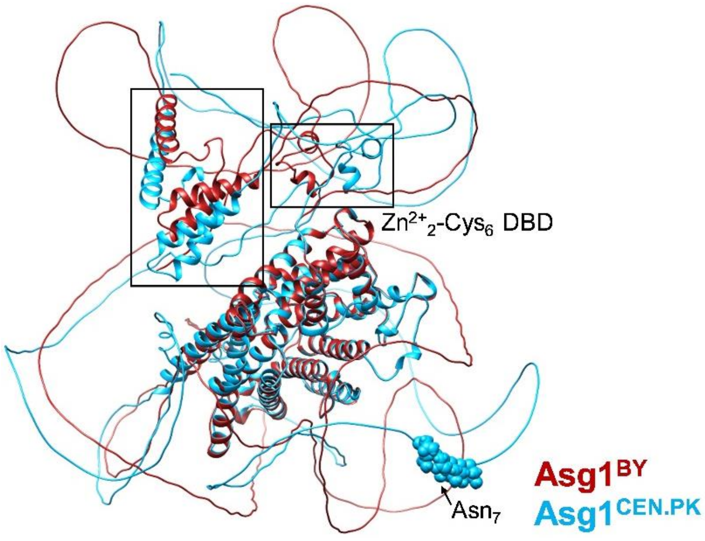

Supplementary Figure 4: **Asg1 structure prediction.** Protein variants of the Asg1 transcription factor were modelled by AlphaFold2 (Jumper et al. 2021) tool and the predicted structures of Asg1^{BY} and Asg1$^{CEN.PK}$ are aligned and presented in red and blue ribbons, respectively. A major primary structure difference of the homologues was observed at position 869 where Asg1^{BY} variant lacks 7 consecutive Asn residues (ball model), but this motif and the surrounding region were presumed not structured and highly disordered. The core of the Asg1 protein was predicted with high certainty where both variants were aligned well. Differences in alignment of structured motifs are marked in rectangles where both α-helices at positions 120-183 and $Zn^{2+}{}_2$-Cys_6 fungal-type DNA-binding domains at positions 21-47 were predicted with high certainty.

Supplementary Table 1: **Primers used for strain reconstruction to suit the backcrossing procedure.**

Name	Sequence	Length of PCR product	Purpose
HIS3del_f	AAGAATATACTAAAAAATGAGCAGGCAAGATAAACGAAGGCAAAGTGACACCGATTATTTAAAGCTGCAGCATACGATATATATACATGT	90 nt	Donor DNA for *HIS3* gene deletion
HIS3del_r	ACATGTATATATATCGTATGCTGCAGCTTTAAATAATCGGTGTCACTTTGCCTTCGTTTATCTTGCCTGCTCATTTTTTAGTATATTCTT		
UP_HIS3_2f	GCATTAGTCAGGGAAGTCAT	1292 bp (wt), 629 bp (deletion)	Control PCR for *HIS3* gene deletion
DN_HIS3_2r	GAGGAACATAACCATTCTCG		
MATa_f	ACTCCACTTCAAGTAAGAGTTTG	544 bp (*MAT*a)	Control PCR for yeast mating type (Illuxley et al. 1990)
MAT_r	AGTCACATCAAGATCGTTTATGG		
MATalpha_f	GCACGGAATATGGGACTACTTCG	404 bp (*MAT*α)	Control PCR for yeast mating type (Illuxley et al. 1990)
MAT_r	AGTCACATCAAGATCGTTTATGG		

Supplementary Table 2: **Primers used for *ENA* locus detection, deletion and swap.**

Name	Sequence	Length of PCR product	Purpose
ENA1_UP_f	GGTCTTTTCCTCTTATTCCGTTCTT	3752 bp (wt BY)	*ENA1* detection within BY *ENA* locus
ENA12_DN_r	CGCGTAACGTAACTGTATCTGTTTC		
ENA25_UP_f	GGTGTAATTGTGAGGCTGATGTTT	3722 bp (wt BY)	*ENA2* detection within BY *ENA* locus
ENA12_DN_r	CGCGTAACGTAACTGTATCTGTTTC		
ENA25_UP_f	GGTGTAATTGTGAGGCTGATGTTT	3783 bp (wt BY)	*ENA5* detection within BY *ENA* locus
ENA5_DN_r	GACCGACTCCAACACCATTAGA		
ENA1_UP_f	GGTCTTTTCCTCTTATTCCGTTCTT	3821 bp (wt CEN.PK)	*ENA6* detection within CEN.PK *ENA* locus
ENA6_r	GAAGCCTGATGTCTTGAGAGAGG		
kan_ENA125_f	CAATACGTATATTTACTAATTAAAGAAAAAACTTCGTACACAGAATTGAAAATTTTCGGACATGGAGGCCCAGAATACC	1473 bp	Donor DNA for *ENA125* deletion within BY locus
kan_ENA125_r	AGGGGGAGAAGGGATAAGGGATGCAAAAGGAAAGGCACTCAATAAATTGCCCTCCTTACAGTATAGCGACCAGCATTCACATAC		
kan_ENA6_f	CAATACGTATATTTACTAATTAAAGAAAAAACTTCGTACACAGAATTGAAAATTTCCGGACATGGAGGCCCAGAATACC	1473 bp	Donor DNA for *ENA6* deletion within CEN.PK locus
kan_ENA6_r	AATATATGATGAAATGGAGTAAGGAAAAAGATAGGGAACACATAATAGGCCCTGCCTACAGTATAGCGACCAGCATTCACATAC		
ins_ENA6_f	CAATACGTATATTTACTAATTAAAGAAAAAACTTCGTACACAGAATTGAAAATTTTCGATGGGCGAAGGAACTACTAAGG	3392 bp	Donor DNA for *ENA125* swap into ENA6
ins_ENA6_r	AGGGGGAGAAGGGATAAGGGATGCAAAAGGAAAGGCACTCAATAAATTGCCCTCCTTATCATTGTTTGATACCAATATTAATTTCTGTATGGATTG		
ins_ENA6_f_short	CAATACGTATATTTACTAATTAAAGAAAAAACTTCG	3386 bp	Donor DNA for *ENA125* swap into *ENA6*
ins_ENA6_r_short	AGAAGGGATAAGGGATGCAAAA		
ENA1_UP_f	GGTCTTTTCCTCTTATTCCGTTCTT	3785 bp (*ENA6*), 1866 bp (deletion)	Control PCR for *ENA6* detection within CEN.PK background
ENA6_r	GAAGCCTGATGTCTTGAGAGAGG		
ENA1_UP_f	GGTCTTTTCCTCTTATTCCGTTCTT	3813 bp (*ENA6*), 1896 bp (*ENA125* deletion)	Control PCR for *ENA125* detection within BY background
ENA5_DN_r	GACCGACTCCAACACCATTAGA		
ins_ENA6_f_short	CAATACGTATATTTACTAATTAAAGAAAAAACTTCG	3386 bp (*ENA6*), 1467 bp (*ENA* deletion)	Control PCR for *ENA* locus detection
ins_ENA6_r_short	AGAAGGGATAAGGGATGCAAAA		

Supplementary Table 3: **Primers used for deletion and allele swap of *ASG1*, *AYR1*, *KGD1*, *MET18*, *QDR2* and *RRT14* genes.**

Name	Sequence	Length of PCR product	Purpose
ASG1_kan_del_f	CCATAAGGTAAGAGACAAAGAAAAAGGAGCGCATATTATAATTGATAAGGGCGTTGACATGGAGGCCCAGAATACC	1467 bp	Donor DNA for *ASG1* deletion
ASG1_kan_del_r	GTTCAGCAAGTGCTGTAGCCACGATATTATAGAATATAAATCAAAGATAACGTTTCAGTATAGCGACCAGCATTCACATAC		
ASG1_UP_f	TTGAAGACAACCAGTAATCGAA	3244 bp (wt), 1769 bp (deletion)	Control PCR for *ASG1*
ASG1_DN_r	AACTAGATCCAGGATTGGTACTA		
ASG1_ins_f	GAGTTCCATAAGGTAAGAGACAAAGAAAAAGGAGCGCATATTATAATTGATAAGGGCGTTATGCCAGAACAAGCGCAACAA	3036 bp	Donor DNA for *ASG1* allele swap
ASG1_ins_r	CTTATGTTCAGCAAGTGCTGTAGCCACGATATTATAGAATATAAATCAAAGATAACGTTTTCATTCAGAGGGGTAATTTAAAGGTAGGTAT		
ASG1_ins_f_short	GAGTTCCATAAGGTAAGAGACAA	3036 bp	Donor DNA for *ASG1* allele swap
ASG1_ins_r_short	CTTATGTTCAGCAAGTGCTGTA		
AYR1_kan_BYdel_f	CCCAACTTCTATTAATATATACTCAATTCAAATATTGATCAGGATTTCAAAGAGTGACATGGAGGCCCAGAATACC	1467 bp	Donor DNA for *AYR1* deletion in BY strain
AYR1_kan_del_r	AAACTATACGGAATCTTGTATATAAATATAGGTAGCTATTGACGATGGTGAAACACAGTATAGCGACCAGCATTCACATAC		
AYR1_kan_CENPKdel_f	TGCCCAACTTCTATTAATATATACTCAATTCAAATATTGATCAGGATTTCAAAGAGTGACATGGAGGCCCAGAATACC	1467 bp	Donor DNA for *AYR1* deletion in CEN.PK strain
AYR1_kan_del_r	AAACTATACGGAATCTTGTATATAAATATAGGTAGCTATTGACGATGGTGAAACACAGTATAGCGACCAGCATTCACATAC		
AYR1_UP_f	TTCAAGAATCATTCTTTACTTTATATTGATAT	1251 bp (wt), 1714 bp (deletion)	Control PCR for *AYR1*
AYR1_DN_r	ACTTAAAGAGCAGTTGAACATTA		
AYR1_BY_ins_f	CATCATGCCCAACTTCTATTAATATACTCAATTCAAATATTGATCAGGATTTCAAAGAGTATGTCGGAGTTACAGTCACAAC	1014 bp	Donor DNA for *AYR1* swap of the BY allele
AYR1_ins_r_short	TGTACAAACTATACGGAATCTTGTATATAA		
AYR1_CENPK_ins_f	TCATGCCCAACTTCTATTAATATATACTCAATTCAAATATTGATCAGGATTTCAAAGAGTATGTCGGAGTTACAGTCACAAC	1014 bp	Donor DNA for *AYR1* swap of the CEN.PK allele
AYR1_ins_r_short	TGTACAAACTATACGGAATCTTGTATATAA		
AYR1_ins_f_short	CATCATGCCCAACTTCTATTAATAT	1014 bp	Donor DNA for *AYR1* allele swap
AYR1_ins_r_short	TGTACAAACTATACGGAATCTTGTATATAA		
KGD1_kan_del_f	AGAAACAACAAAAGAGAAAGAAAGCAGTTTTTAGAAAATACTAAATTTTACCGTTGACATGGAGGCCCAGAATACC	1467 bp	Donor DNA for *KGD1* deletion
KGD1_kan_del_r	AATATTACGTGTTTATATGGCTGTATTTTCATGTTTTTCATATTTG		

	AATTCATCTCAGTATAGCGACCAGCATTCACATAC		
KGD1_UP_f	GAAGGAAGCGATTCTCGTTC	3335 bp (wt), 1647 bp (deletion)	Control PCR for *KGD1*
KGD1_DN_r	AAGACTAAGCAGATGCTAACATCAT		
KGD1_ins_f_short	ATAGTTTCGTTTGAAAGAAACAAC	3196 bp	Donor DNA for *KGD1* allele swap
KGD1_ins_r_short	AGGCTGTCCATAAACATTAAGG		
MET18_by_kan_del_f	TAGTTTTCATTGAACTTGTTTTAACTGGGAAAAAGCGGAACAATTGGGCCTTACAGACATGGAGGCCCAGAATACC	1467 bp	Donor DNA for *MET18* deletion in BY strain
MET18_kan_del_r	CTCGCTAAGATTTTCACGTGCTCATCAATGTGAACAAATTATTAAATACAAGCGTCAGTATAGCGACCAGCATTCACATAC		
MET18_cenpk_kan_del_f	TAATTTTCATTGAACTTGTTTTAACTGGGAAAAAGCGGAACAATTGGGACTTACAGACATGGAGGCCCAGAATACC	1467 bp	Donor DNA for *MET18* deletion in CEN.PK strain
MET18_kan_del_r	CTCGCTAAGATTTTCACGTGCTCATCAATGTGAACAAATTATTAAATACAAGCGTCAGTATAGCGACCAGCATTCACATAC		
MET18_UP_f	GGAGTCACCTCTATGATCATC	3236 bp (wt), 1494 bp (deletion)	Control PCR for *MET18*
MET18_DN_r	GATTTTCACGTGCTCATCAATG		
MET18_BY_ins_f	GCTGTTAATTTTCATTGAACTTGTTTTAACTGGGAAAAAGCGGAACAATTGGGACTTACAATGACACCAGACGAACTAAATTCAG	3223 bp	Donor DNA for *MET18* swap of the BY allele
MET18_ins_r_short	CTTAGTATACTCGCTAAGATTTTCACG		
MET18_CENPK_ins_f	GCTGTTAGTTTTCATTGAACTTGTTTTAACTGGGAAAAAGCGGAACAATTGGGCCTTACAATGACACCAGACGAACTAAATTCAG	3223 bp	Donor DNA for *MET18* swap of the CEN.PK allele
MET18_ins_r_short	CTTAGTATACTCGCTAAGATTTTCACG		
MET18_BY_ins_f_short	GCTGTTAATTTTCATTGAACTTGTTT	3223 bp	Donor DNA for *MET18* swap of the BY allele
MET18_ins_r_short	CTTAGTATACTCGCTAAGATTTTCACG		
MET18_CENPK_ins_f_short	GCTGTTAGTTTTCATTGAACTTGTTT	3223 bp	Donor DNA for *MET18* swap of the CEN.PK allele
MET18_ins_r_short	CTTAGTATACTCGCTAAGATTTTCACG		
QDR2_kan_BYdel_f	TCAGTTGCTGGTCATTTTAGTAGAAACTCTGCTCTCAAACTTGAGTACTGCAACGGACATGGAGGCCCAGAATACC	1467 bp	Donor DNA for *QDR2* deletion in BY strain
QDR2_kan_BYdel_r	CGGTAGTGGAGCGATCAAAAGGAACATTTTCCTTTGATTCAAGAAGCTTTACTTCCAGTATAGCGACCAGCATTCACATAC		
QDR2_kan_CENPKdel_f	AGCAACATCAAGTATAATTCGGGAAAATGATTTTGAGGACGAGCTTGCGGAGCGTGACATGGAGGCCCAGAATACC	1467 bp	Donor DNA for *QDR2* deletion in CEN.PK strain
QDR2_kan_CENPKdel_r	GAGTTCAGATTAAAAAAAAAAATACTTACTATTACTTTCTATTTGAGAAGACTTACAGTATAGCGACCAGCATTCACATAC		
QDR2_UP_f	GTCACCATCTTTTCCTAGATAAAGA	1958 bp (wt), 1744 bp (deletion)	Control PCR for *QDR2*
QDR2_DN_r	CAAAACTCAGTACTAGCCGTT		
QDR2_BY_ins_f	GCAGGAGCAACATCAAGTATAATTCGGGAAAATGATTTTGAGGACGAGCTTGCGGAGCGTATGGCAGGAGCAACATCAAGTATAATT	1749 bp	Donor DNA for *QDR2* swap of the BY allele
QDR2_BY_ins_r	TTACAGAGTTCAGATTAAAAAAAAAATACTTACTATTACTTTCT		

	ATTTGAGAAGACTTATTAATTTACTCCCAGTTCTTGCTTTTTCCTCTTAA		
QDR2_BY_ins_f_short	GCAGGAGCAACATCAAGTATAATT	1764 bp	Donor DNA for *QDR2* swap of the BY allele
QDR2_BY_ins_r_short	CTCTAATTTTAGTTATTACAGAGTTCAGATT		
QDR2_CENPK_ins_f	GCAAGTCAGTTGCTGGTCATTTTAGTAGAAACTCTGCTCTCAAACTTGAGTACTGCAACGATGCAGTCATATAATAGAGAAACTGCAGAC	1749 bp	Donor DNA for *QDR2* swap of the CEN.PK allele
QDR2_CENPK_ins_r	TTTCTCGGTAGTGGAGCGATCAAAAGGAACATTTTCCTTTGATTCAAGAAGCTTTACTTCTCACACCAATTCCTCTTTCTCGGTAGT		
QDR2_CENPK_ins_f_short	GCAAGTCAGTTGCTGGTCATTTTAG	1749 bp	Donor DNA for *QDR2* swap of the CEN.PK allele
QDR2_CENPK_ins_r_short	TTTCTCGGTAGTGGAGCGATC		
RRT14_kan_del_f	GTGGAAAAGTTGTTTAGAATACGAACAACTTAAGGAGGGAGCTAGCTAGGCCAAAGACATGGAGGCCCAGAATACC	1467 bp	Donor DNA for *RRT14* deletion in BY strain
RRT14_by_kan_del_r	TAAATATGATTCTTTTATGTATAGTACAGCAAAATAGCATTTATTTTTATTACGTCAGTATAGCGACCAGCATTCACATAC		
RRT14_kan_del_f	GTGGAAAAGTTGTTTAGAATACGAACAACTTAAGGAGGGAGCTAGCTAGGCCAAAGACATGGAGGCCCAGAATACC	1467 bp	Donor DNA for *RRT14* deletion in CEN.PK strain
RRT14_cenpk_kan_del_r	TAAATATGATTCTTTTATGTATAGTACAGCAAAATAGCATTTAATTTTATTACGTCAGTATAGCGACCAGCATTCACATAC		
RRT14_UP_f	TGGAAATATAGCTGGCAACTG	811 bp (wt), 1547 bp (deletion)	Control PCR for *RRT14*
RRT14_DN_r	GTTCACATTGATGAGCACGTG		
RRT14_ins_f_short	TATAGCTGGCAACTGTGGAAATATTG	766 bp	Donor DNA for *RRT14* swap of the BY allele
RRT14_BY_ins_r	CTAAGTAAATATGATTCTTTTATGTATAGTACAGCAAAATAGCATTTAATTTTATTACGTTTAATCTTCTTCACTAGAGTCTTCCTCG		
RRT14_ins_f_short	TATAGCTGGCAACTGTGGAAATATTG	766 bp	Donor DNA for *RRT14* swap of the CEN.PK allele
RRT14_CENPK_ins_r	CTAAGTAAATATGATTCTTTTATGTATAGTACAGCAAAATAGCATTTATTTTTATTACGTTTAATCTTCTTCACTAGAGTCTTCCTCG		
RRT14_ins_f_short	TATAGCTGGCAACTGTGGAAATATTG	766 bp	Donor DNA for *RRT14* allele swap
RRT14_ins_r_short	CTAAGTAAATATGATTCTTTTATGTATAGTACAGC		

Supplementary Table 4: **Amino acids composition for sporulation medium** (Pačnik et al. 2021)**.** The sporulation medium contains 0.25% yeast extract, 1.50% KAc, 0.25% glucose, and indicated amino acids selection.

Amino acid	Final concentration (mg/l)	Source
adenine	4	#A14906, Alfa Aesar, USA
arginine	2	#W381918, SAFC, USA
histidine	2	#H6034, Sigma Aldrich, Germany
leucine	2	#27690, Serva, Germany
lysine	2	#28195, Serva, Germany
metionine	2	#166160250, Acros Organics, USA
phenylalanine	10	#32191, Serva, Germany
threonine	25	#36382, Serva, Germany
tryptophan	2	#37422, Serva, Germany
tyrosine	4	#37540, Serva, Germany
uracil	4	#U0750, Sigma Aldrich, Germany

Supplementary Table 5: **Amino acids composition for spore selection medium** (Tong and Boone 2006)**.** The spore selection medium contains 6.9 g/l YNB medium without amino acids (yeast nitrogen base), 2% glucose, 60 mg/l canavanine, 60 mg/l thialysine, and indicated amino acids selection.

Amino acid	Final concentration (mg/l)	Source
adenine	122	#A14906, Alfa Aesar, USA
alanine	82	#11482, Serva, Germany
asparagine	82	#A-4284, Sigma Aldrich, Germany
aspartate	82	#105045000, Thermo Scientific, UK
cysteine	82	#C7352, Sigma Aldrich, Germany
glutamate	82	#G1626, Sigma Aldrich, Germany
glutamine	82	#22930, Serva, Germany
glycine	82	#33226, Sigma Aldrich, Germany
inositol	82	#57570, Fluka, Germany
isoleucine	82	#I7403, Sigma Aldrich, Germany
leucine	408	#27690, Serva, Germany
methionine	82	#166160250, Acros Organics, USA
phenylalanine	82	#32191, Serva, Germany
proline	82	#7430, Merck, Germany
serine	82	#S4500, Sigma Aldrich, Germany
threonine	82	#36382, Serva, Germany
tryptophan	82	#37422, Serva, Germany
tyrosine	82	#37540, Serva, Germany
uracil	82	#U0750, Sigma Aldrich, Germany
valine	82	#94619, Fluka, Germany

Supplementary Table 6: **Primers used for construction of gRNA expression plasmid.** The expressed gRNA targeted KanMX resistance marker, while the expression plasmid had a hygromycin (hph) expression marker.

Name	Sequence	Length of PCR product	Purpose
p426_BB_KanMX_f	ATGAAGGAGAAAACTCACCGGTTTTAGAGCTAGAAATAGCAAGTTAAAATAA	4017 bp	Fragment for gRNA incorporation into p426 URA3 plasmid, with overhangs for Gibson assembly
p426_backbone_r	TTTCACCAGCGTTTCTGGGTGAGCAAAAAACAGGAAGGCAA		
p426_insert_f	TTGCCTTCCTGTTTTTGCTCACCCAGAAACGCTGGTGAAA	2349 bp	Fragment for gRNA incorporation into p426 KanMXR plasmid, with overhangs for Gibson assembly
p426_ins_KanMX_r	TTATTTTAACTTGCTATTTCTAGCTCTAAAACCGGTGAGTTTTCTCCTTCATGATCATTTATCTTTCACTGCGGAGAAGTTTCG		
hph_p426_f	GTGAGTTTAGTATACATGCATTTACTTATAATACAGTTTTTAAAGCCTTCGAGCGTCC	1722 bp	Fragment of hph resistance marker insert, with overhangs for Gibson assembly
hph_p426_r	TTCTCACATCACATCCGAACATAAACAACCATGGGTAAAAAGCCTGAACTCAC		
p426_beforeR_r	AAACTGTATTATAAGTAAATGCATGTATACTAAACTCAC	5242 bp	Fragment of p426 gRNA plasmid backbone without resistance marker, with overhangs for Gibson assembly
p426_afterR_f	AAGGTGGTATGGTGCACTCT		

Supplementary Table 7: **List of plasmids used in the study.**

Name	Properties	Source
p414	Expression of SpCas9 endonuclease under strong constitutive TEF1 promoter, NatMX selection marker	(DiCarlo et al. 2013; Pačnik et al. 2021)
p426 gHIS3	Expression of gRNA targeting HIS3 gene, KanMX selection marker	(Žun et al. 2022)
p426 gKan	Expression of gRNA, targeting KanMX marker gene, hph (hygromycin) selection marker	this study
pHS3	Expression of HO endonuclease for mating type switch, NatMX selection marker	#81038, Addgene

Supplementary Table 8: **List of potential QTGs.** The table presents a list of predicted QTLs for NaCl salt tolerance, including their position, size and contained genes. The prediction is based on F1 generation segregant pool by the QTLseqr tool (Mansfeld and Grumet 2018), complementary presented on the Supplementary Figure 1 in File 1. The assessed genes are marked in bold.

QTL	Chr	Coordinates	List of genes
**QTL1**	**chrIV**	503,000-655,000	YDR030C (RAD28), YDR031W (MIX14), YDR032C (PST2), YDR033W (MRH1), YDR034C (LYS14), YDR034C-A, YDR034W-B, YDR035W (ARO3), YDR036C (EHD3), YDR037W (KRS1), **YDR038C (ENA5), YDR039C (ENA2), YDR040C (ENA1),** YDR041W (RSM10), YDR042C, YDR043C (NRG1), YDR044W (HEM13), YDR045C (RPC11), YDR046C (BAP3), YDR047W (HEM12), YDR048C, YDR049W (VMS1), YDR050C (TPI1), YDR051C (DET1), YDR052C (DBF4), YDR053W, YDR054C (CDC34), YDR055W (PST1), YDR056C (EMC10), YDR057W (YOS9), YDR058C (TGL2), YDR059C (UBC5), YDR060W (MAK21), YDR061W, YDR062W (LCB2), YDR063W (AIM7), YDR064W (RPS13), YDR065W (RRG1), YDR066C (RTR2), YDR067C (OCA6), YDR068W (DOS2), YDR069C (DOA4), YDR070C (FMP16), YDR071C (PAA1), YDR072C (IPT1), YDR073W (SNF11), YDR074W (TPS2), YDR075W (PPH3), YDR076W (RAD55), YDR077W (SED1), YDR078C (SHU2), YDR079W (PET100), YDR079C-A (TFB5), YDR080W (VPS41), YDR081C (PDC2), YDR082W (STN1), YDR083W (RRP8), YDR084C (TVP23), YDR085C (AFR1), YDR086C (SSS1), YDR087C (RRP1), YDR088C (SLU7), YDR089W (VTC5), YDR090C, YDR091C (RLI1), YDR092W (UBC13), YDR093W (DNF2), YDR094W, YDR095C, YDR096W (GIS1), YDR097C (MSH6), YDR098C (GRX3), YDR099W (BMH2)
**QTL2**	**chrV**	64,000-110,000	YEL049W (PAU2), YEL048C (TCA17), YEL047C, YEL046C (GLY1), YEL045C, YEL044W (IES6), YEL043W, YEL042W (GDA1), YEL041W (YEF1), YEL040W (UTR2), YEL039C (CYC7), YEL038W (UTR4), YEL037C (RAD23), YEL036C (ANP1), YEL035C (UTR5), YEL034C-A, YEL034W (HYP2), YEL033W (MTC7), YEL032W (MCM3), YEL032C-A, YEL031W (SPF1), YEL030C-A, YEL030W (ECM10), YEL029C (BUD16), YEL028W, YEL027W (VMA3), YEL026W (SNU13), YEL025C, YEL024W (RIP1), YEL023C
**QTL3**	**chrV**	179,000-230,000	YER013W (PRP22), YER014W (HEM14), YER014C-A (BUD25), YER015W (FAA2), YER016W (BIM1), YER017C (AFG3), YER018C (SPC25), YER019W (ISC1), YER019C-A (SBH2), YER020W (GPA2), YER021W (RPN3), YER022W (SRB4), YER023W (PRO3), YER023C-A, YER024W (YAT2), YER025W (GCD11), YER026C (CHO1), YER027C (GAL83), YER028C (MIG3), YER029C (SMB1), YER030W (CHZ1), YER031C (YPT31), YER032W (FIR1), YER033C (ZRG8), YER034W, YER035W (EDC2), YER036C (ARB1), YER037W (PHM8), YER038C (KRE29), YER038W-A (FMP49), YER039C (HVG1), YER039C-A, YER040W (GLN3
**QTL4**	**chrVI**	92,000-209,000	YFL023W (BUD27), YFL022C (FRS2), YFL021C-A, YFL021W (GAT1), YFL020C (PAU5), YFL019C, YFL018C (LPD1), YFL017W-A (SMX2), YFL017C (GNA1), YFL016C (MDJ1), YFL015W-A, YFL015C, YFL014W (HSP12), YFL013W-A, YFL013C (IES1), YFL012W-A, YFL012W, YFL011W (HXT10), YFL010W-A (AUA1), YFL010C (WWM1), YFL009W (CDC4), YFL008W (SMC1), YFL007W (BLM10), YFL005W (SEC4), YFL004W (VTC2), YFL003C (MSH4), YFL002C (SPB4), YFL001W (DEG1), YFR001W (LOC1), YFR002W (NIC96), YFR003C (YPI1), YFR004W (RPN11), YFR005C (SAD1), YFR006W, YFR007W

			(YFH7), YFR008W (FAR7), YFR009W (GCN20), YFR009W-A, YFR010W (UBP6), YFR010W-A, YFR011C, YFR012W (DCV1), YFR012W-A, YFR013W (IOC3), YFR014C (CMK1), YFR015C (GSY1), YFR016C, YFR017C (IGD1), YFR018C, YFR019W (FAB1), YFR020W (CSS2), YFR021W (ATG18), YFR022W (ROG3), YFR023W (PES4), YFR024C-A (LSB3), YFR025C (HIS2), YFR026C (ULI1), YFR027W (ECO1), YFR028C (CDC14)
**QTL5**	**chrVII**	208,000-280,000	YGL158W (RCK1), YGL157W (ARI1), YGL156W (AMS1), YGL155W (CDC43), YGL154C (LYS5), YGL153W (PEX14), YGL152C, YGL151W (NUT1), YGL150C (INO80), YGL149W, YGL148W (ARO2), YGL147C (RPL9A), YGL146C (RRT6), YGL145W (TIP20), YGL144C (ROG1), YGL143C (MRF1), YGL142C (GPI10), YGL141W (HUL5), YGL140C, YGL139W (FLC3), YGL138C, YGL137W (SEC27), YGL136C (MRM2), YGL135W (RPL1B), YGL134W (PCL10), YGL133W (ITC1), YGL132W, YGL131C (SNT2), YGL130W (CEG1), YGL129C (RSM23), YGL128C (CWC23), YGL127C (SOH1), YGL126W (SCS3), YGL125W (MET13), YGL124C (MON1), YGL123W (RPS2), YGL123C-A, YGL122C (NAB2)
**QTL6**	**chrVII**	221,000-272,000	YGL150C (INO80), YGL149W, YGL148W (ARO2), YGL147C (RPL9A), YGL146C (RRT6), YGL145W (TIP20), YGL144C (ROG1), YGL143C (MRF1), YGL142C (GPI10), YGL141W (HUL5), YGL140C, YGL139W (FLC3), YGL138C, YGL137W (SEC27), YGL136C (MRM2), YGL135W (RPL1B), YGL134W (PCL10), YGL133W (ITC1), YGL132W, YGL131C (SNT2), YGL130W (CEG1), YGL129C (RSM23), YGL128C (CWC23), YGL127C (SOH1), YGL126W (SCS3
**QTL7**	**chrIX**	2,000-94,000	YIL177C, YIL176C (PAU14), YIL173W (VTH1), YIL172C (IMA3), YIL171W-A, YIL169C, YIL166C, YIL165C, YIL164C (NIT1), YIL163C, YIL162W (SUC2), YIL161W, YIL160C (POT1), YIL159W (BNR1), YIL158W (AIM20), YIL157C (COA1), YIL156W-A, YIL156W-B, YIL156W (UBP7), YIL155C (GUT2), YIL154C, YIL153W (RRD1), YIL152W, YIL151C (ESL1), YIL150C (MCM10), YIL149C (MLP2), YIL148W (RPL40A), YIL147C (SLN1), YIL146C (ATG32), YIL145C (PAN6), YIL144W, YIL143C (SSL2), YIL142C-A, YIL142W (CCT2), YIL141W, YIL140W (AXL2), YIL139C (REV7), YIL138C (TPM2), YIL137C (TMA108), YIL136W (OM45)
**QTL8**	**chrIX**	97,000-134,000	YIL134W (FLX1), YIL133C (RPL16A), YIL132C (CSM2), YIL131C (FKH1), **YIL130W (ASG1)**, YIL129C (TAO3), **YIL128W (MET18)**, **YIL127C (RRT14)**, YIL126W (STH1), **YIL125W (KGD1)**, **YIL124W (AYR1)**, YIL123W (SIM1), YIL122W (POG1), **YIL121W (QDR2)**
**QTL9**	**chrIX**	243,000-276,000	YIL063C (YRB2), YIL062C (ARC15), YIL061C (SNP1), YIL060W, YIL059C, YIL058W, YIL057C (RGI2), YIL056W (VHR1), YIL055C, YIL054W, YIL053W, YIL052C (RPL34B), YIL051C (MMF1), YIL050W (PCL7), YIL049W (DFG10), YIL048W (NEO1), YIL047C-A, YIL047C (SYG1), YIL046W-A, YIL046W (MET30), YIL045W (PIG2), YIL044C (AGE2), YIL043C (CBR1), YIL042C (PKP1)
**QTL10**	**chrXI**	62,000-80,000	YKL203C (TOR2), YKL202W, YKL201C (MNN4), YKL198C (PTK1), YKL197C (PEX1), YKL196C (YKT6), YKL195W (MIA40), YKL194C (MST1), YKL193C (SDS22)
**QTL11**	**chrXI**	132,000-141,000	YKL168C (KKQ8), YKL167C (MRP49), YKL166C (TPK3), YKL165C-A, YKL165C (MCD4)
**QTL12**	**chrXI**	560,000-616,000	YKR062W (TFA2), YKR063C (LAS1), YKR064W (OAF3), YKR065C (PAM17), YKR066C (CCP1), YKR067W (GPT2), YKR068C (BET3), YKR069W (MET1), YKR070W, YKR071C (DRE2), YKR072C (SIS2), YKR073C, YKR074W (AIM29), YKR075C, YKR075W-A, YKR076W

			(ECM4), YKR077W (MSA2), YKR078W, YKR079C (TRZ1), YKR080W (MTD1), YKR081C (RPF2), YKR082W (NUP133), YKR083C (DAD2), YKR084C (HBS1), YKR085C (MRPL20), YKR086W (PRP16), YKR087C (OMA1), YKR088C (TVP38), YKR089C (TGL4), YKR090W (PXL1), YKR091W (SRL3), YKR092C (SRP40), YKR093W (PTR2)
**QTL13**	**chrXII**	409,000-450,000	YLR133W (CKI1), YLR134W (PDC5), YLR135W (SLX4), YLR136C (TIS11), YLR137W (RKM5), YLR138W (NHA1), YLR139C (SLS1), YLR140W, YLR141W (RRN5), YLR142W (PUT1), YLR143W (DPH6), YLR144C (ACF2), YLR145W (RMP1), YLR146C (SPE4), YLR146W-A, YLR147C (SMD3), YLR148W (PEP3), YLR149C, YLR149C-A, YLR150W (STM1), YLR151C (PCD1), YLR152C, YLR153C (ACS2), YLR154C (RNH203)
**QTL14**	**chrXII**	482,000-520,000	YLR157W-E, YLR157C-C, YLR158C (ASP3-3), YLR159W, YLR159C-A, YLR160C (ASP3-4), YLR161W, YLR162W, YLR162W-A (RRT15), YLR163C (MAS1), YLR163W-A, YLR164W (SHH4), YLR165C (PUS5), YLR166C (SEC10), YLR167W (RPS31), YLR168C (UPS2), YLR169W, YLR170C (APS1), YLR171W, YLR172C (DPH5), YLR173W, YLR174W (IDP2), YLR175W (CBF5), YLR176C (RFX1), YLR177W, YLR178C (TFS1), YLR179C, YLR180W (SAM1), YLR181C (VTA1), YLR182W (SWI6)
**QTL15**	**chrXII**	556,000-586,000	YLR206W (ENT2), YLR207W (HRD3), YLR208W (SEC13), YLR209C (PNP1), YLR210W (CLB4), YLR211C (ATG38), YLR212C (TUB4), YLR213C (CRR1), YLR214W (FRE1), YLR215C (CDC123), YLR216C (CPR6), YLR217W, YLR218C (COA4), YLR219W (MSC3), YLR220W (CCC1), YLR221C (RSA3), YLR222C (UTP13), YLR222C-A, YLR223C (IFH1)
**QTL16**	**chrXII**	820,000-883,000	YLR344W (RPL26A), YLR345W, YLR346C (CIS1), YLR347C (KAP95), YLR347W-A, YLR348C (DIC1), YLR349W, YLR350W (ORM2), YLR351C (NIT3), YLR352W, YLR353W (BUD8), YLR354C (TAL1), YLR355C (ILV5), YLR356W (ATG33), YLR357W (RSC2), YLR358C, YLR359W (ADE13), YLR360W (VPS38), YLR361C (DCR2), YLR361C-A, YLR362W (STE11), YLR363C (NMD4), YLR363W-A, YLR364W (GRX8), YLR365W, YLR364C-A, YLR366W, YLR367W (RPS22B), YLR368W (MDM30), YLR369W (SSQ1), YLR370C (ARC18), YLR371W (ROM2), YLR372W, YLR373C (VID22), YLR374C, YLR375W (STP3), YLR376C (PSY3), YLR377C (FBP1), YLR378C (SEC61), YLR379W, YLR380W (CSR1), YLR381W (CTF3), YLR382C (NAM2)
**QTL17**	**chrXII**	889,000-910,000	YLR384C (IKI3), YLR385C (SWC7), YLR386W (VAC14), YLR387C (REH1), YLR388W (RPS29A), YLR389C (STE23), YLR390W (ECM19), YLR390W-A (CCW14), YLR392C (ART10), YLR393W (ATP10), YLR394W (CST9), YLR395C (COX8)
**QTL18**	**chrXII**	932,000-968,000	YLR406C-A, YLR407W, YLR408C (BLS1), YLR409C (UTP21), YLR410W (VIP1), YLR411W (CTR3), YLR412W (BER1), YLR412C-A, YLR413W (INA1), YLR414C (PUN1), YLR415C, YLR416C, YLR417W (VPS36), YLR418C (CDC73), YLR419W, YLR420W (URA4), YLR421C (RPN13), YLR422W (DCK1)
**QTL19**	**chrXIII**	800,000-880,000	YMR266W (RSN1), YMR267W (PPA2), YMR268C (PRP24), YMR269W (TMA23), YMR270C (RRN9), YMR271C (URA10), YMR272C (SCS7), YMR272W-A, YMR272W-B, YMR273C (ZDS1), YMR274C (RCE1), YMR275C (BUL1), YMR276W (DSK2), YMR277W (FCP1), YMR278W (PRM15), YMR279C, YMR280C (CAT8), YMR281W (GPI12), YMR282C (AEP2), YMR283C (RIT1), YMR284W (YKU70), YMR285C (NGL2), YMR286W (MRPL33), YMR287C (DSS1), YMR288W (HSH155),

			YMR289W (ABZ2), YMR290C (HAS1), YMR290W-A, YMR291W (TDA1), YMR292W (GOT1), YMR293C (HER2), YMR294W (JNM1), YMR294W-A, YMR295C, YMR296C (LCB1), YMR297W (PRC1), YMR298W (LIP1), YMR299C (DYN3), YMR300C (ADE4), YMR301C (ATM1), YMR302C (YME2), YMR303C (ADH2), YMR304W (UBP15), YMR304C-A, YMR305C (SCW10)
**QTL20**	**chrXIII**	916,000-951,000	YMR320W, YMR321C, YMR322C (SNO4), YMR323W (ERR3), YMR324C, YMR325W (PAU19), YMR326C
**QTL21**	**chrXIV**	320,000-388,000	YNL167C (SKO1), YNL166C (BNI5), YNL165W, YNL164C (IBD2), YNL163C (RIA1), YNL162W-A, YNL162W (RPL42A), YNL161W (CBK1), YNL160W (YGP1), YNL159C (ASI2), YNL158W (PGA1), YNL157W (IGO1), YNL156C (NSG2), YNL155W (CUZ1), YNL154C (YCK2), YNL153C (GIM3), YNL152W (INN1), YNL151C (RPC31), YNL150W, YNL149C (PGA2), YNL148C (ALF1), YNL147W (LSM7), YNL146C-A, YNL146W, YNL145W (MFA2), YNL144C, YNL144W-A, YNL143C, YNL142W (MEP2), YNL141W (AAH1), YNL140C, YNL139C (THO2), YNL138W-A (YSF3), YNL138W (SRV2), YNL137C (NAM9), YNL136W (EAF7), YNL135C (FPR1), YNL134C, YNL133C (FYV6), YNL132W (KRE33), YNL131W (TOM22), YNL130C (CPT1), YNL130C-A (DGR1), YNL129W (NRK1), YNL128W (TEP1), YNL127W (FAR11), YNL126W (SPC98)
**QTL22**	**chrXV**	254,000-306,000	YOL039W (RPP2A), YOL038C-A, YOL038W (PRE6), YOL037C, YOL036W, YOL035C, YOL034W (SMC5), YOL033W (MSE1), YOL032W (OPI10), YOL031C (SIL1), YOL030W (GAS5), YOL029C, YOL028C (YAP7), YOL027C (MDM38), YOL026C (MIM1), YOL025W (LAG2), YOL024W, YOL023W (IFM1), YOL022C (TSR4), YOL021C (DIS3), YOL020W (TAT2), YOL019W-A, YOL019W, YOL018C (TLG2), YOL017W (ESC8), YOL016C (CMK2), YOL015W (IRC10), YOL014W, YOL013W-B, YOL013W-A, YOL013C (HRD1), YOL012C (HTZ1), YOL011W (PLB3)

Supplementary references

DiCarlo JE et al. 2013. Genome engineering in Saccharomyces cerevisiae using CRISPR-Cas systems. Nucleic acids research. 41(7):4336–4343

Illuxley C, Green ED, Dunbam I. 1990. Rapid assessment of S. cerevisiae mating type by PCR. Trends in Genetics. 6(8):236. https://doi.org/10.1016/0168-9525(90)90190-H

Jumper J et al. 2021. Highly accurate protein structure prediction with AlphaFold. Nature. 596(7873). https://doi.org/10.1038/s41586-021-03819-2

Mansfeld BN, Grumet R. 2018. QTLseqr: An R Package for Bulk Segregant Analysis with Next-Generation Sequencing. The Plant Genome. 11(2). https://doi.org/10.3835/plantgenome2018.01.0006

Pačnik K et al. 2021. Identification of novel genes involved in neutral lipid storage by quantitative trait loci analysis of Saccharomyces cerevisiae. BMC Genomics. 22(1). https://doi.org/10.1186/s12864-021-07417-4

Tong AHY, Boone C. 2006. Synthetic Genetic Array Analysis in Saccharomyces cerevisiae. In: Yeast Protocols. https://doi.org/10.1016/S0076-6879(10)70007-0

Žun G, Doberšek K, Petrovič U. 2022. Construction and Evaluation of gRNA Arrays for Multiplex CRISPR-Cas9. Yeast (Chichester, England). (July 2022):32–41 http://www.ncbi.nlm.nih.gov/pubmed/36536407. https://doi.org/10.1002/yea.3833
